# Traffic exhaust to wildfires: PM_2.5_ measurements with fixed and portable, low-cost LoRaWAN-connected sensors

**DOI:** 10.1371/journal.pone.0231778

**Published:** 2020-04-24

**Authors:** Hugh Forehead, Johan Barthelemy, Bilal Arshad, Nicolas Verstaevel, Owen Price, Pascal Perez

**Affiliations:** 1 SMART Infrastructure Facility, University of Wollongong, Wollongong, Australia; 2 Clean Air and Urban Landscapes (CAUL) hub, Melbourne, Victoria, Australia; 3 Université Toulouse 1 Capitole, Institut de Recherche en Informatique de Toulouse (IRIT), Toulouse, France; 4 Centre for Sustainable Ecosystem Solutions, University of Wollongong, Wollongong, Australia; Universidade de Vigo, SPAIN

## Abstract

Air pollution with PM_2.5_ (particulate matter smaller than 2.5 micro-metres in diameter) is a major health hazard in many cities worldwide, but since measuring instruments have traditionally been expensive, monitoring sites are rare and generally show only background concentrations. With the advent of low-cost, wirelessly connected sensors, air quality measurements are increasingly being made in places where many people spend time and pollution is much worse: on streets near traffic. In the interests of enabling members of the public to measure the air that they breathe, we took an open-source approach to designing a device for measuring PM_2.5_. Parts are relatively cheap, but of good quality and can be easily found in electronics or hardware stores, or on-line. Software is open source and the free LoRaWAN-based “The Things Network” the platform. A number of low-cost sensors we tested had problems, but those selected performed well when co-located with reference-quality instruments. A network of the devices was deployed in an urban centre, yielding valuable data for an extended time. Concentrations of PM_2.5_ at street level were often ten times worse than at air quality stations. The devices and network offer the opportunity for measurements in locations that concern the public.

## 1. Introduction

### 1.1. The health hazard

Airborne pollution by fine particles (PM_2.5_) is increasingly recognised as a serious hazard to health [1]. There are harmful effects to brain function, the heart, circulatory system [2], respiratory system [3], immune system, endocrine system [4] and to unborn foetuses [5]. Airborne particulate matter is classified by the nominal diameter of the particles in microns or micrometres (μm or 10^−6^ m, the diameter of an average human hair is about 75 microns), for example: PM_2.5_ is particles with aerodynamic diameters smaller than 2.5 microns (10^−6^ m or 1/1000 of a milimetre) and PM_10_, 10 microns. PM is harmful because it generally comprises a mixture of toxic compounds (in rare cases there may be a single ingredient), solid and liquid, that can be inhaled into the lungs. The particles can become trapped in the lining of the lung, where toxins can be efficiently transferred to sensitive tissues and into the blood stream. The finer the particles, the deeper they can be inhaled into the lungs. The finest fraction that is commonly measured, studied and regulated is PM_2.5_. Ultrafine particles, PM_1_ and even PM_0.1_ increasingly feature in studies; but the difficulty of measuring at that scale continues to limit research and regulation [6–9].

### 1.2. Measurements

Publicly available measurements of PM_2.5_, where they exist, are generally of background levels in selected areas within cities [10]. The reason for this is usually just economics; the reference grade equipment for measuring air quality is expensive [11], so that installations are rare and end up being far apart. Data from the instruments are used to give an averaged level of pollution across wide areas [10]. These networks are usually operated by government agencies and are the main source of information about urban air quality for the public. Concentrations of pollutants from traffic are, of course, greatest near busy roads [12, 13]. This is where many people in cities spend much of their time: commuting, at work, or shopping. Measurements of air quality in these places are rare and usually intermittent [13].

Particulate matter from vehicle emissions is difficult to measure because of the composition of the particles (solid, liquid, or a combination of the two) and because particle size and number can vary significantly over time and space. Some PM, called ‘primary’ PM, is released directly from the exhaust pipe; others, called ‘secondary’ particles, are created and change size as gases condense and particles aggregate. Smoke from the burning of vegetation, whether from anthropogenic or natural causes, is another significant source of particulate pollution in many parts of the world; the range of compounds and of particle sizes involved can be large [14]. These fires are also increasing in frequency with climate change [15]. There are several methods of measuring PM and in some cases, two different methods may yield different results for the same sample [8]. The reference method pumps air through a filter that traps PM; the pore size of the filter determines the size range of the particles. Other technologies include tapered element oscillating microbalances (TEOM), beta attenuation monitors (BAM) and light scattering monitors. The last of these is currently the simplest and cheapest to manufacture [16, 17].

### 1.3. Air quality and IoT

The recent increase in the availability of cheap, low-powered sensors, combined with free, open radio networks (such as The Things Network, TTN) has led to a boom in measurements of many environmental parameters, including air quality. This technology, commonly called the Internet of Things (IoT), makes possible the deployment of sensors in many more locations than in the past. The technology offers the potential for non-scientists to make their own devices and measurements and to make the data publicly available. However, there can be significant problems with the reliability and accuracy of data from low-cost air quality instruments [13]. The sensors are of variable quality and the resulting low-cost instruments are often not subject to testing, calibration or oversight by experts in the measurement. Much of the IoT market is filled with suppliers who offer cheap sensors with little or no quality control. Air quality sensors for many parameters are designed for measuring industrially high concentrations of pollutants and do not operate well in ambient conditions. PM concentration is currently one of the most successful air quality measurements being made with IoT sensors [18]. Light scattering sensors offer a relatively stable and reliable method for detecting PM and can be bought for as little as AU$30. However, the quality of manufacture and measurements is usually difficult to assess without buying them to test. Specifications and documentation may not be available, may be out of date, incomplete or unreliable.

### 1.4. Motivation

Air quality was cited as an important concern in consultative public meetings in 2018 in Liverpool, Australia, a municipality that suffered the worst pollution of PM_2.5_ in the state of New South Wales in that year [19]. There has also been significant attention paid in the media to poor air quality from smoke over the greater Sydney region, during nearby hazard reduction burns [20–22] and worldwide coverage following the extensive bushfires of 2019–2020 [23, 24]. Increased monitoring was seen to be a valuable implementation of IoT technology for the community during public meetings.

The aim of the project was to design a trustworthy, adaptable air quality monitoring device that could be constructed and used by any moderately skilled member of the public. The emphasis was on producing measurements that were as accurate and useful as practicable, while keeping the cost of sensors as low as possible. We chose parts that were easily available and could be bought from hardware stores, or on-line. Software was a combination of existing and new open-source libraries and firmware, made freely available by the authors. This paper describes a process of scoping, designing, testing and deploying low-cost air quality sensors.

## 2. Materials and methods

### 2.1. Aim

Particulate matter sensing devices were required for 2 different projects: the first was for roadside measurements of pollution in a busy urban centre and the second for quantifying the smoke produced by forest fires. The housings had to be hardy, weatherproof, easily mounted and resistant to invasion by small animals. The design was intended to be cheap and suitable for construction by the general public, without special tools or equipment.

### 2.2. Design and specifications

#### 2.2.1. Variations

There were 4 variations to the design of the device, all using the same PM sensor:

- stationary, power—240 V AC, data—LoRaWAN network
- mobile, power—battery (5 V), data—LoRaWAN network
- mobile, power—battery (5 V), data—SD card, internal clock
- mobile, power—battery (5 V), data—SD card, geolocation & timekeeping—GPS

**Fig 1:**
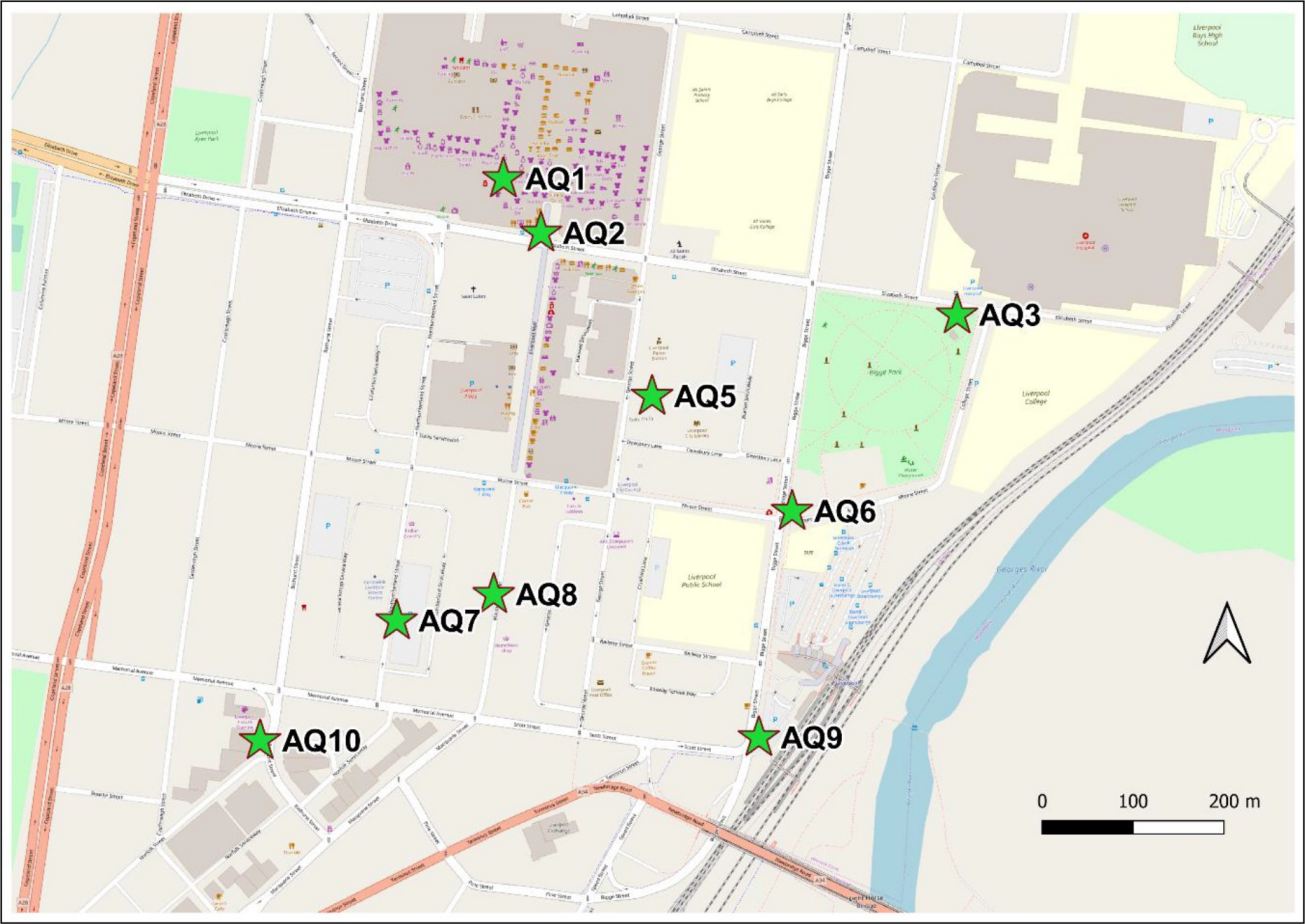
Map of the city centre of Liverpool, New South Wales showing locations of permanently installed air quality devices (AQxx). Liverpool air quality monitoring station is 1.35 km WSW from lower left corner of map. Base map: Open Street Map, 2020.

The first versions of the sensors sent data over free and long-range wireless networks in urban settings. The devices were designed to be situated outdoors in a busy city centre and data were collected via The Things Network. We deployed free LoRaWAN networks, well suited for IoT applications, in Wollongong and Liverpool, New South Wales. Liverpool is a rapidly growing centre in the south-west of Sydney, Australia [25]. The municipality includes the construction site for Sydney’s new second airport. Wollongong is a coastal city of just over 302,000 people [25,26], 68 km south of Sydney. Mains AC power (240 V) was available for the sensors that were installed in city centres (Fig 1). Portable sensors were built to be powered with batteries, for mobile roadside measurements. Timestamping of the collected data is managed by The Things Network. Sensors for measuring smoke in remote areas had to run on batteries (5 V) for 48 h. Since no network was available, data were written to SD memory cards. The sensors operated independently, making measurements at a distance around the perimeter of the fire, so the readings needed accurate timestamps. Stationary units had an on-board clock with a drift of +/- 30 sec over 48 h. Portable devices kept time with satellite data from their Global Positioning System (GPS) integrated module, also used for geolocation.

#### 2.2.2. Housing

**Fig 2:**
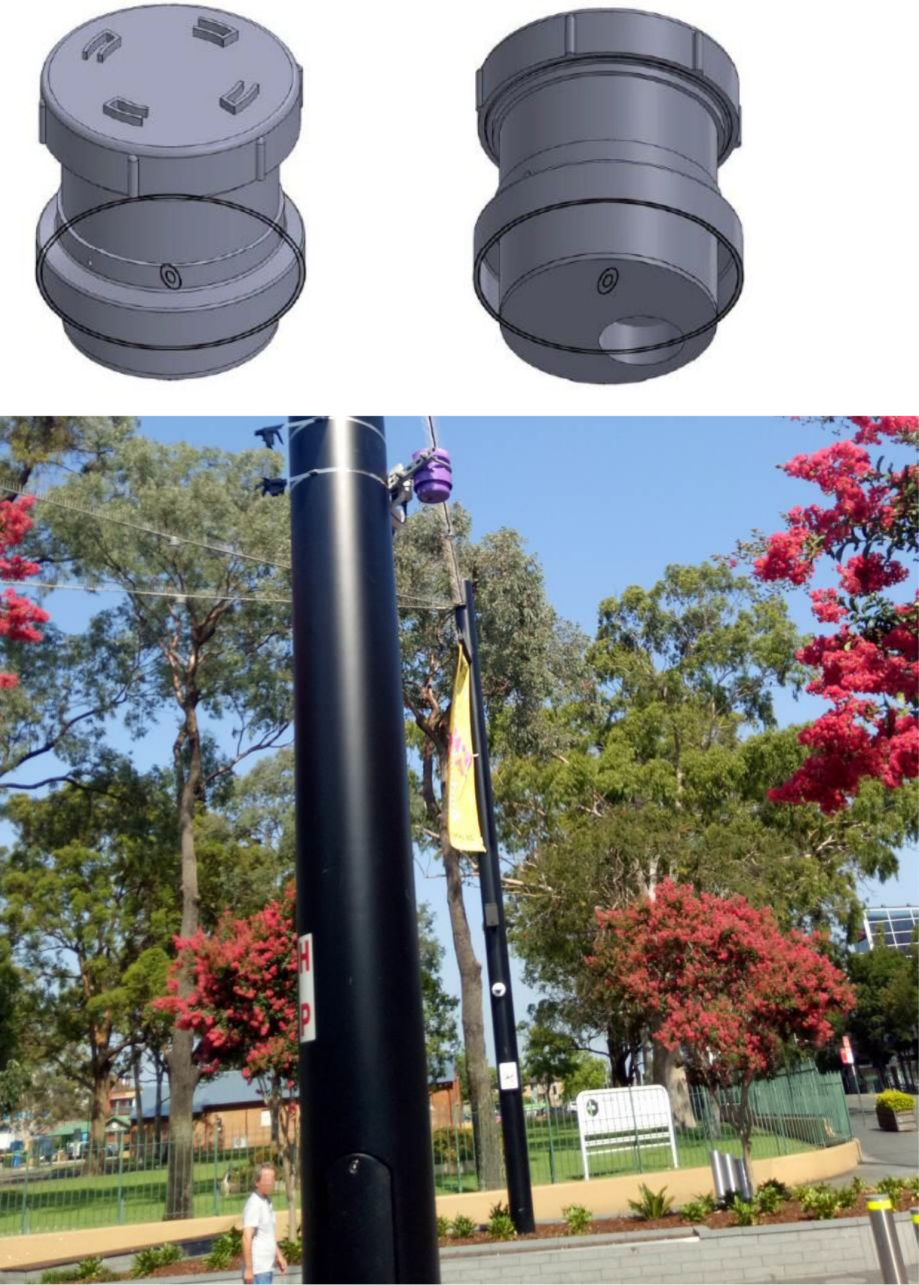
Housing of the air quality device (top images) and installed (purple coloured) on pole in Liverpool Mall (images: top, S. Selby; bottom, H. Forehead).

The housing (Fig 2) was built almost entirely from 100 mm diameter PVC plumbing parts, with a few screws and small pieces of fibreglass insect screen. Overall outside dimensions were 130 mm in diameter and 140 mm high See S1 Appendix for details of parts and instructions for assembly. The cost of the parts for the housing was about AU$16. The lid had a waterproof o-ring seal and was 100 mm diameter, allowing easy access. Air was circulated through the housing with a 5 V DC, 30 mm diameter fan. The air inlets were multiple (sheltered) 12 mm diameter holes around the sides, the outlet was a recessed opening in the base of the housing. All the openings were protected from entry by pests with insect screening. The housing was divided into upper and lower sections by a platform, used to mount the electronics and keep those parts away from the air flow. The housing could be mounted with a standard 100 mm pipe bracket from a hardware or plumbing store.

#### 2.2.3. Controllers

Two different controller platforms were used for the roadside and remote settings. The LoPy 4 [27], manufactured by PyCom Ltd (Guildford, UK), and the open source Arduino Uno [28]. LoPy4 was a good choice for when LoRaWAN network connectivity was available. It combines, on a single integrated circuit, radio-communications (LoRaWAN, Bluetooth Low Energy, WiFi and Sigfox) capacity with a micro-controller. Programming for the LoPy4 is in MicroPython [29], a variation of the popular Python [30] language. The LoPy development board [31] provides an SD card socket and a small aerial was attached inside the case. Arduino hardware is cheaper than the LoPy4 and works well in situations where radio-communications were not available. The language is C++ and there are many software libraries available. More recent versions of Arduino have LoRaWAN capability built in (e.g. Seeed studio). Software created for the devices is open source and freely available for download. The devices with Arduino processors used Arduino scripts and the devices equipped with LoPy4 chips used Micropython. The firmware is freely available [32], the code is under an MIT License, compatible with the GPL License; it is free software and complies with the best standards of scientific reproducibility.

#### 2.2.4. Sensors

There are many low-cost sensors advertised as suitable for measuring particulate matter. We tested 3 different brands of units before settling on the Nova SDS011. It has a larger air circulating fan than other units, giving a greater sampling volume. The manufacturers claim a detection limit of 0.3 μm. BJHike HK-A5 initially performed consistently, 2 units in the same housing gave readings in excellent agreement. However, sensitivity was poor, with readings under-estimating concentrations by about 80%. Two of these devices failed (only gave readings of 0) after only 6 weeks of use. Plantower branded sensors PMS5003-T and PMS6003 were found to have inlet & outlet ports adjacent to each other, on the same face of the housing. Due to the design of the device housing, it was not practicable to build a partition to prevent cross-flow between these. The sensors were not used because of the risk of inaccuracy related to the potential for feedback.

#### 2.2.5. Other hardware

Power was supplied with either a 5 v DC, 20,000 mAh generic USB power bank, or with a 240 V AC to 6 A, 5 V DC transformer (Mean Well MPM-30-5). The real-time clock unit first used (RTC12) was found to perform poorly, varying considerably over short deployments. It was replaced with a more accurate unit (Linker RTC XC4584). An Arduino-based GPS shield (Adafruit Ultimate GPS Logger Shield #1272) with an SD card socket was used for geolocation and timekeeping in the portable smoke-measuring devices.

### 2.3. Deployments

In the description that follows, the Novasense Nova SDS011 unit will be referred to as the sensor and the complete machine (sensor, processor and other parts) in the housing, as the device. Two different types of devices were constructed around the sensors; one for mobile operation, the other for fixed installations.

One of these devices was fitted with 2 sensors for testing, to check for the repeatability of measurements between sensors. The device was in the grounds of Bluescope Steel at Port Kembla NSW for 14 days in August 2018. The site gave a wide range of values, from near background to severe pollution, according to wind direction and speed. In previous tests, it was found that significantly different readings were made by sensors located about 30 cm apart in different housings. This was most likely due to spatial heterogeneity of the PM_2.5_. It was hoped that locating 2 sensors in the same housing would remove most of the heterogeneity found in open air.

**Fig 3:**
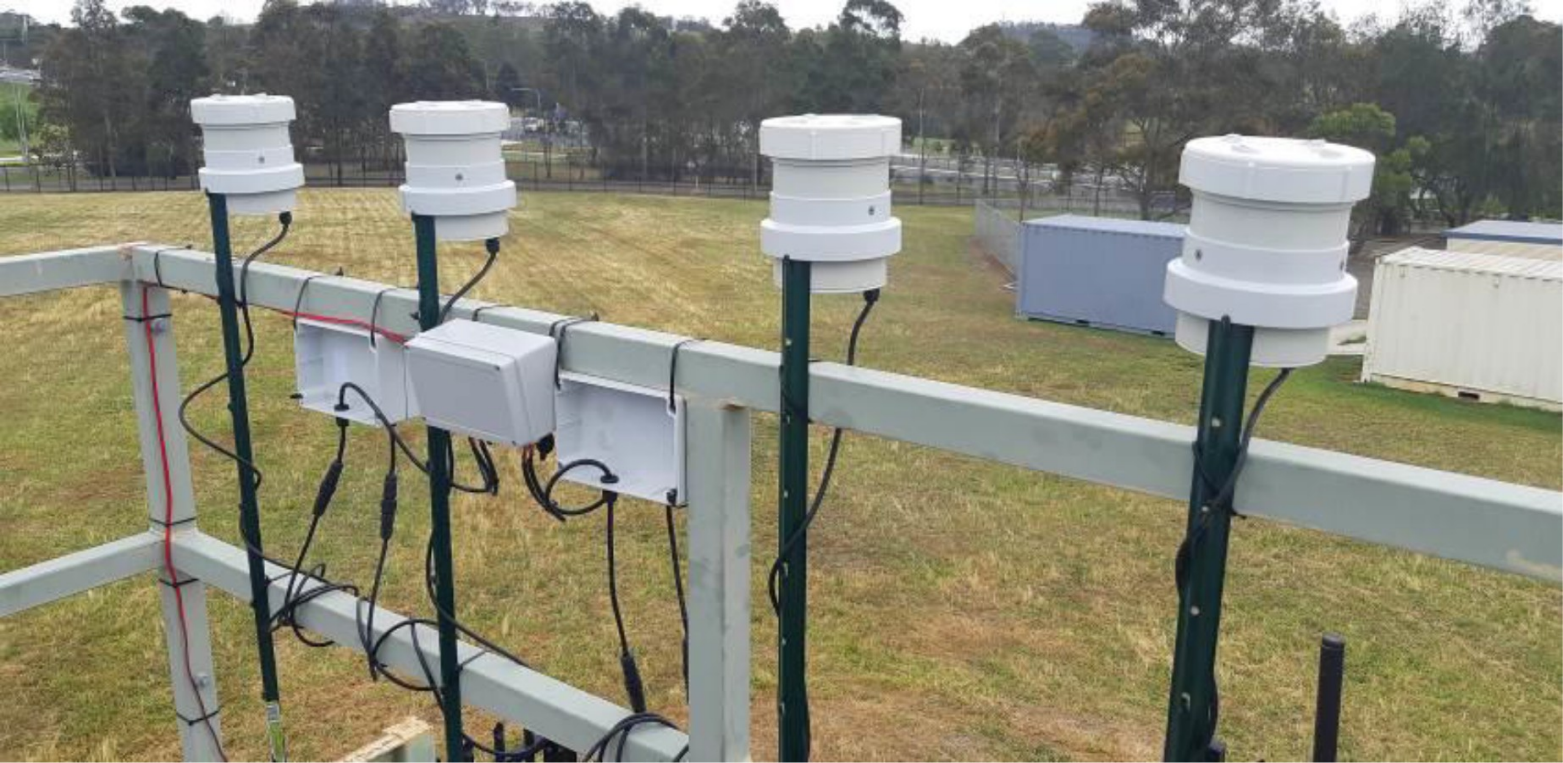
PM measuring devices under test at Department of Planning, Industry and Environment (DPIE) Campbelltown West air quality monitoring station (image: O. Price).

Measurements to validate performance of the devices (Fig 3), were made over 26 days from mid November 2018 at the Campbelltown West air quality monitoring station (Department of Planning, Industry and Environment, DPIE) at a height of 3 m, about 1 m below the reference equipment. The monitoring station is in the grounds of the TAFE college at Campbelltown, NSW. A second installation for validation was over 2 days in June 2019 at the DPIE air quality station in Beaton Park, Wollongong, NSW. There the low-cost devices were at a height of 2 m, about 2 m below the reference equipment. The DPIE air quality station in Liverpool, NSW was used for comparison to roadside measurements in the city centre. All these facilities used a Beta Attenuation Method (BAM) TEI 5014i/TEI 5030 to measure PM_2.5_ and a Filter Dynamic Measurement System (FDMS) TEOM TEI 1405-DF to measure PM_10_. Measurements for low-cost devices and reference equipment were averaged over 1 hour intervals.

A network of the low-cost monitors was installed in the city centre of Liverpool in March 2019 (Fig 1). Installations were made in 9 locations along roads, limited to where mains power was available on council-owed assets. Monitors were mounted as close as practicable to pedestrian height, on structures such as light poles, shade awnings, or walls. Heights of 4 m to 6 m above the ground were chosen to fit with existing equipment and to deter vandalism. A further 11 monitors were built for installation in the future; in the meanwhile they have been made available for use by local school students.

The Low-cost Air Quality Monitors are also being used by the Bushfire Risk Management Research Hub (University of Wollongong) to measure and map the fine-scale distribution of smoke pollution from prescribed burns in the Sydney region. Mobile measurements are at a height of around 1–1.5 m, depending on fixed or vehicle mounting. This enables fire researchers to explore the reasons why some prescribed burns cause large smoke events. We are working closely with the DPIE and NSW Rural Fire Service to inform their fire management to minimise smoke impact while still protecting life and property from bushfires.

### 2.4. Statistics

Statistical analyses and plots were produced in the R environment [33], with the packages “OpenAir” [34] and “ggplot2” [35].

## 3. Results

### 3.1. Consistency between sensors

Two sensors were compared by locating them in the same housing and making measurements for 14 days.

**Fig 4:**
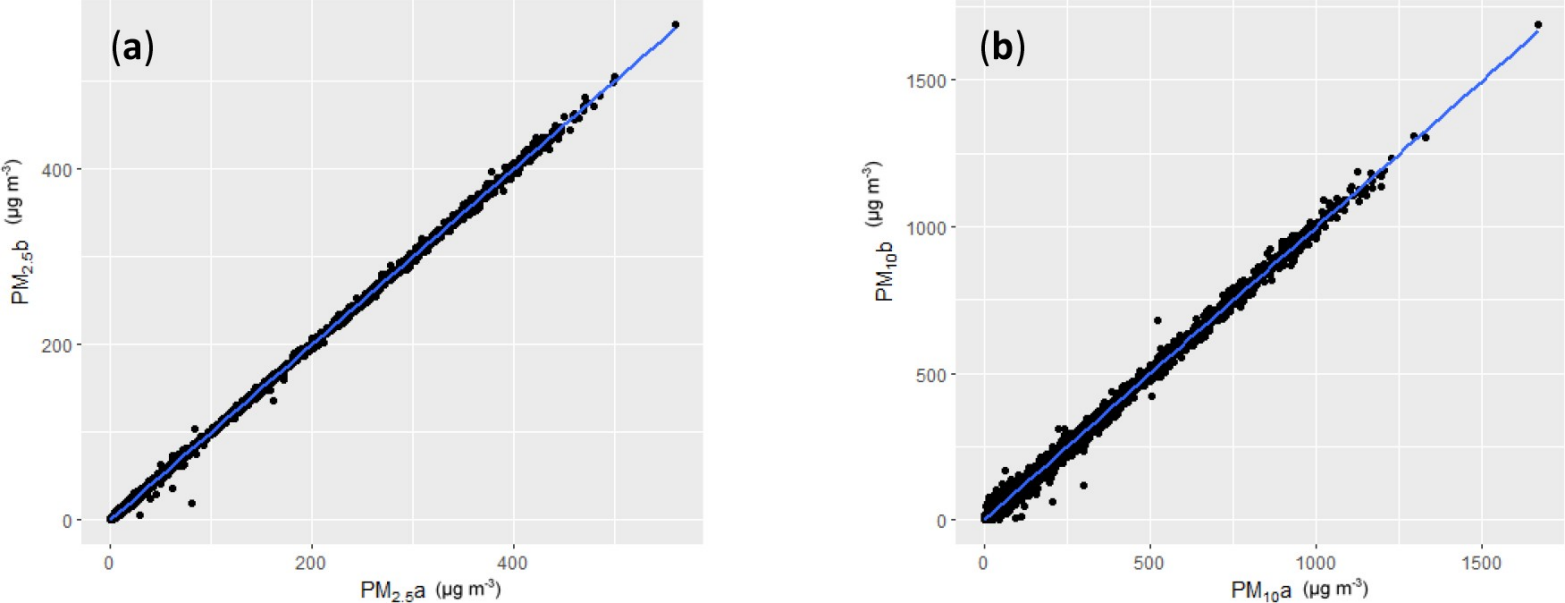
Particulate Matter (PM) measurements (μg m^-3^) from 2 sensors, PMa & PMb, located in the same housing. Raw data taken every 40 seconds for 14 days: a) PM_2.5_, b) PM_10_.

The 2 sensors in the same housing produced very similar readings in the comparison. Data was collected every 40 sec for 14 days, resulting in 30,909 readings. The full dataset is plotted for PM_2.5_ (Fig 4A) and PM_10_ (Fig 4B). The regression equations showed very good agreement between the sensors for both PM_2.5_ and PM_10_:

$$PM_{2.5}b=1.0001\;PM_{2.5}a+0.0095,adjusted\;r^{2}=0.9994,$$

$$PM_{10}b=0.9989\;PM_{10}a+0.1779,adjusted\;r^{2}=0.9958;$$

With averaging over 15 minutes, the small differences in raw data between the sensors were almost eliminated:

$$PM_{2.5}b=1.0004\;PM_{2.5}a+0.00011,adjusted\;r^{2}=1,$$

$$PM_{10}b=1.0011\;PM_{10}a‐0.0190,adjusted\;r^{2}=0.9998$$

A Kruskal-Wallis rank sum test for PM_2.5_ gave χ^2^ = 9.9598e-07 (df = 1, p-value = 0.9992) and for PM_10_, χ^2^ = 8.9638e-06 (df = 1, p-value = 0.9976)

The comparison between sensors in the single housing showed excellent agreement between the 2 sensors across all concentrations measured. The differences in spread between PM_2.5_ and PM_10_ are most likely due to the 3-fold increase in concentration for PM_10_. The small scatter of readings suggests that averaging of the data is beneficial. A 15 minutes interval of averaging gives a good compromise between accuracy and temporal resolution for these sensors.

**Fig 5:**
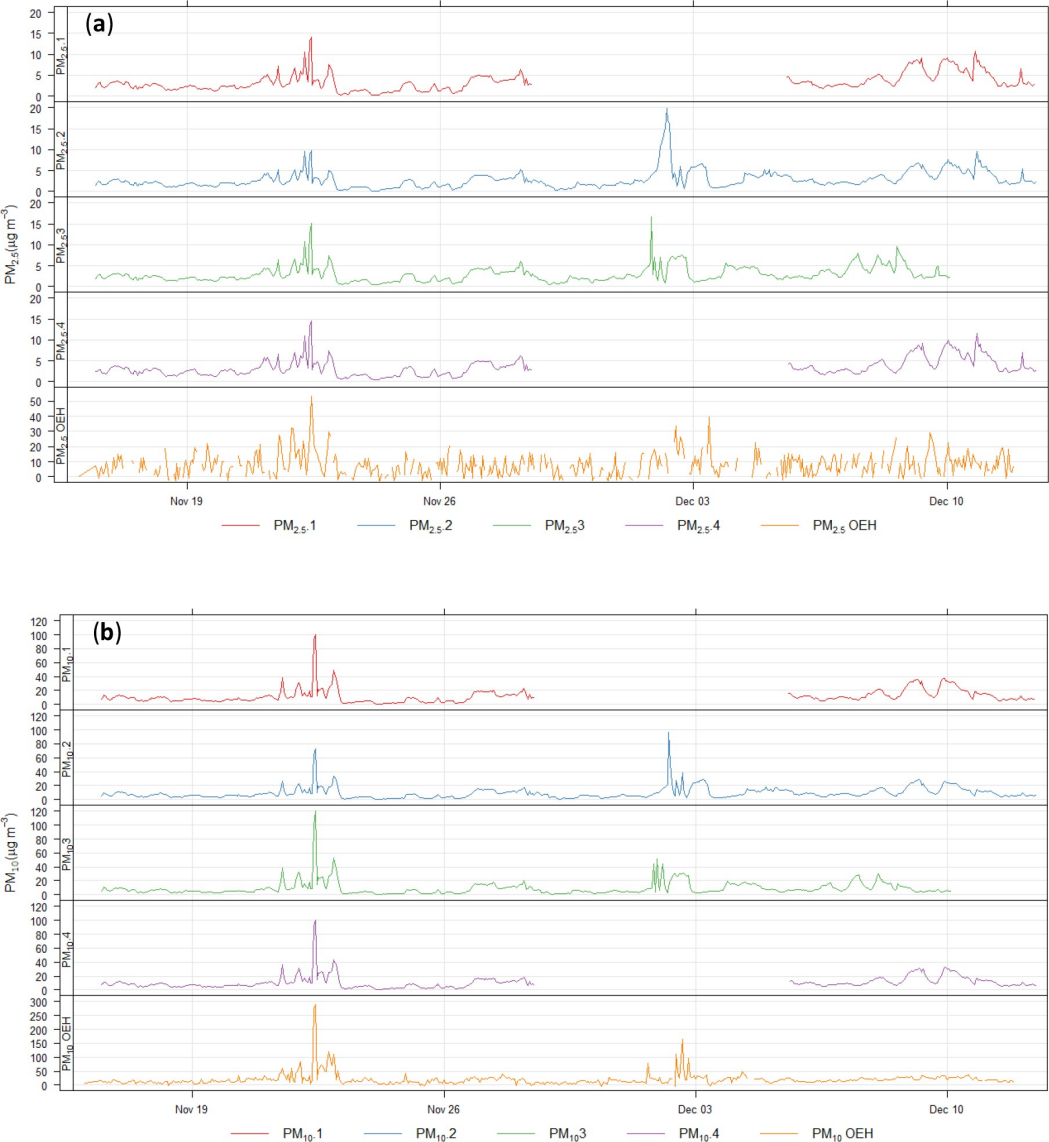
Hourly averaged PM measurements (μg m^-3^, note different scales) from 4 devices co-located with Department of Planning, Industry and Environment (DPIE) reference instrument in December 2018. (a) PM_2.5_ 1–4, PM_2.5_ DPIE and (b) PM_10_ 1–4, PM_10_ DPIE; at DPIE air quality monitoring station in Campbelltown, western Sydney. Missing data in plots 1 & 4 due to power failure, time lag in 3 from early December due to a failing battery in the device’s clock.

The 4 week test at the Campbelltown Air Quality Station (Fig 5) revealed some technical problems, but indicated that the devices produced useful data. Values from the 4 devices were around 1/3 the concentration of the reference instrument, but readings were very consistent between the 4. Peaks of concentration were also detected in a similar manner to the reference instrument, though some detail was lost. Note the large peak in all readings on November 22^nd^, due to a severe dust storm, followed by heavy rain. The omission of higher peak values was most likely due to the 1 h averaging of variable data, required to match the reference instrument. The real-time clock in sensor 3 drifted significantly, leading to an asynchronous trace, though the readings were otherwise similar to the other devices.

This comparison with reference equipment showed that the devices performed well at a range of urban concentrations of particulate matter and could detect elevated concentrations. The accuracy cannot be guaranteed, so caution should be exercised when interpreting the data.

**Fig 6:**
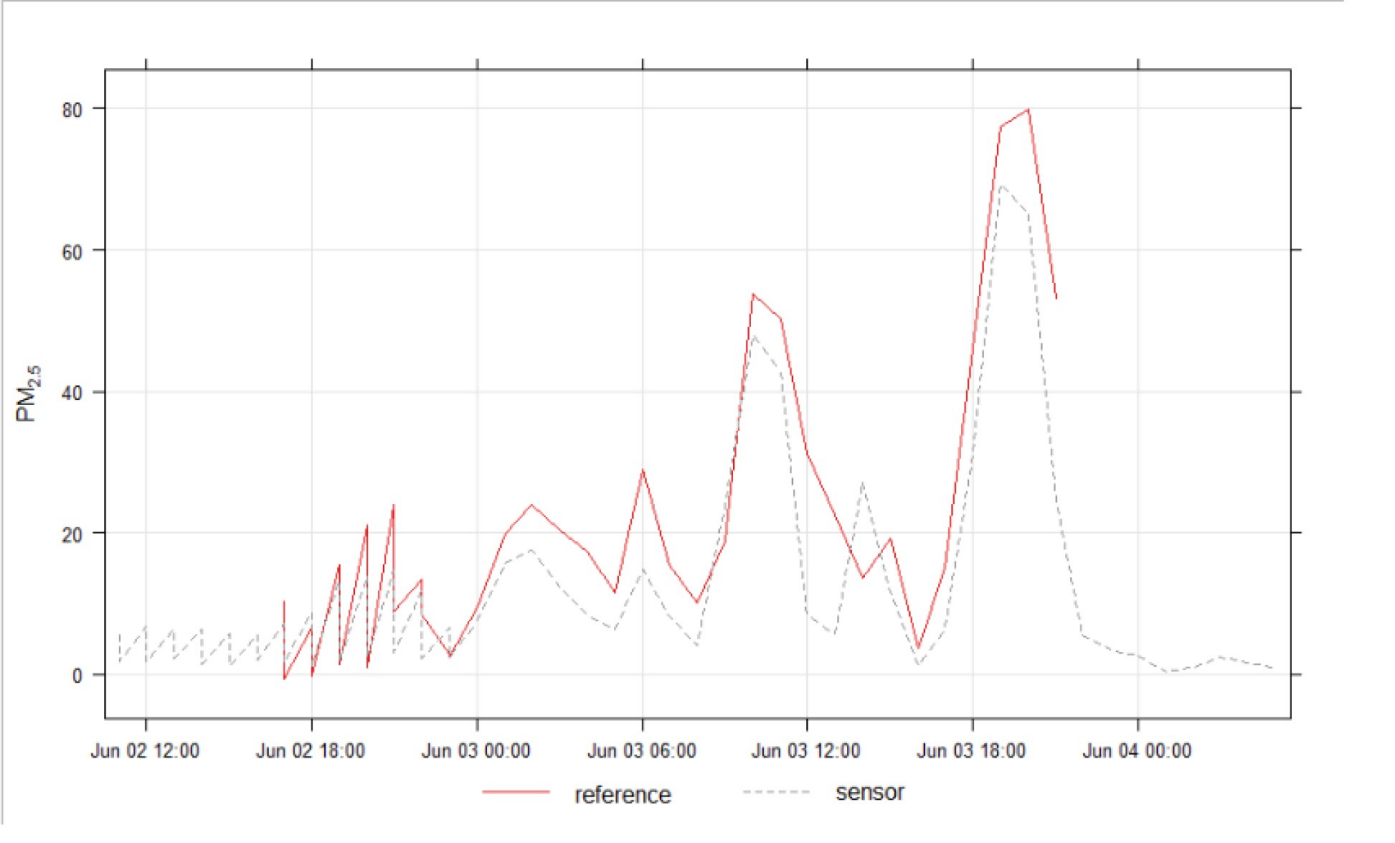
Hourly PM_2.5_ measurements in Wollongong, showing smoke from hazard reduction burn at Avon Dam (approximately 20 km away) 3^rd^ June 2019, showing similarity of readings from low cost device and Department of Planning, Industry and Environment (DPIE) reference equipment.

To test for performance of the devices measuring smoke from bushfires, we co-located a device at an DPIE facility in Wollongong, NSW (Fig 6) during an episode of smoke pollution from a hazard reduction burn at Avon Dam, about 20 km away. The device performed well, capturing peaks of pollution and showing similar values (adjusted R^2^ = 0.8577) to the reference equipment.

These readings demonstrated that the devices were likely to perform well for measuring smoke.

### 3.2. Networked sensors in western Sydney

**Fig 7:**
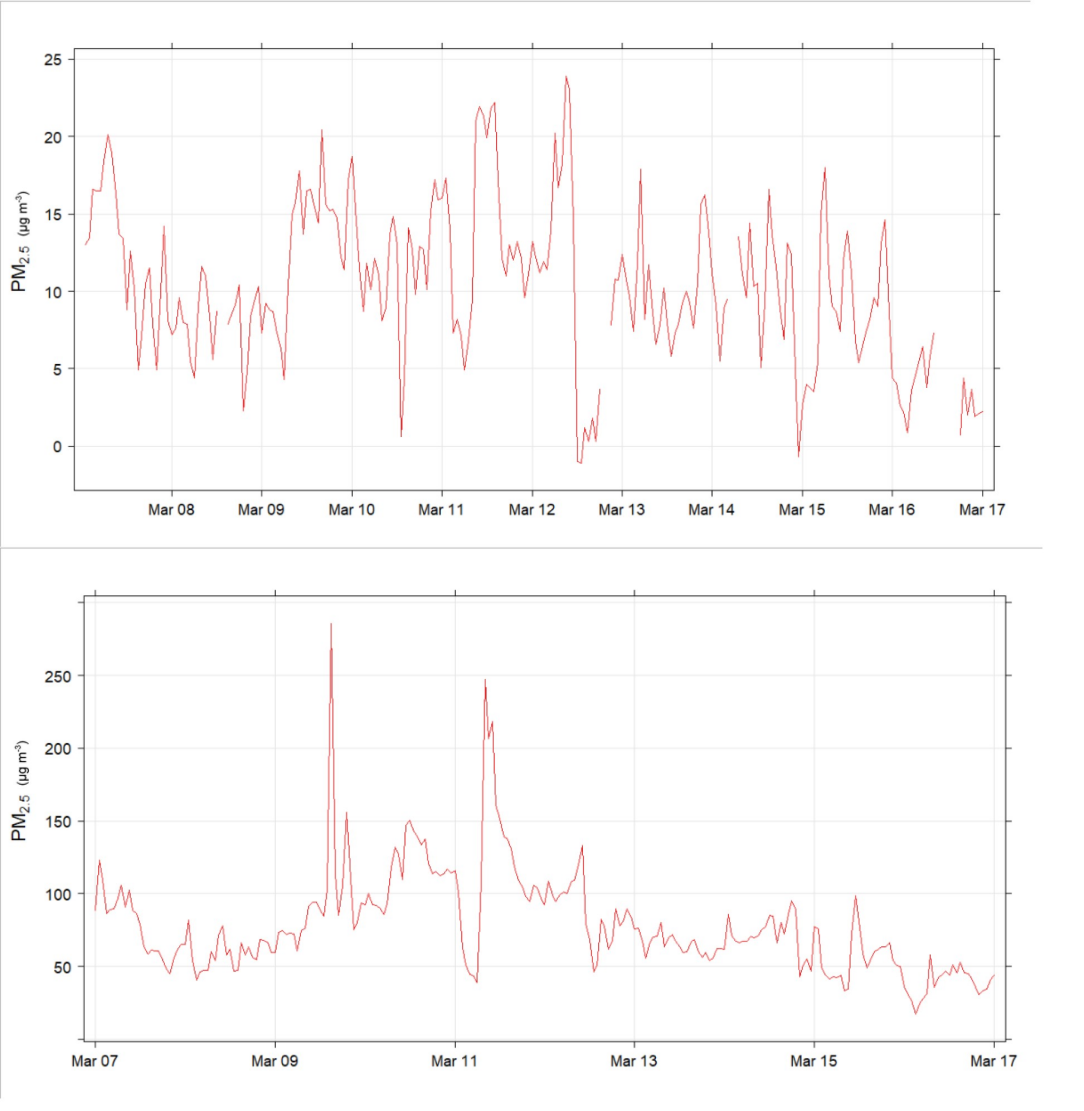
Time series plots showing differences in hourly PM_2.5_ readings (μg m^-3^, note different scales) between an air quality monitoring station and at street-level, over 9 days in March 2019. Averaging period 1 hr. There was no pollution from bushfires in the time shown. (top) Department of Planning, Industry and Environment (DPIE) air quality monitoring station in Pearce Park, Liverpool. (bottom) AQ9 at the corner of Bigge St & Scott St, a ‘T’ intersection near the train station, 1.9 km ENE of Pearce Park.

20 networked PM sensing devices were built and deployed to Liverpool, NSW as part of the Smart Cities and Suburbs Program, Smart Liverpool, Smart Pedestrians project [36]. Fig 1 shows a map of the study area and the fixed air quality devices. Devices installed at the roadside showed substantially elevated readings of PM_2.5_ compared to background air quality figures from the nearest DPIE Air Quality Monitoring Station in Pearce Park, Liverpool (Fig 7). Values at the intersection of Bigge St and Scott St were generally around an order of magnitude greater than background, consistent with more severe pollution caused by stop-start traffic [37–39]. There were few similarities between the 2 time-series, suggesting that the air quality at the roadside was mainly influenced by local pollution, from motor vehicles; as has been found in cities in other countries [40–43]. Roadside values were elevated well above the Australian recommended safe limits (8 μg m^-3^ annual average or 25 μg m^-3^ daily average [44]) for much of the measuring period. This is consistent with the results of a study [45] in Randwick, a municipality in south-eastern Sydney. PM_2.5_ concentrations along a main road there were about double those at the nearby Randwick Air Quality Monitoring Station.

**Fig 8:**
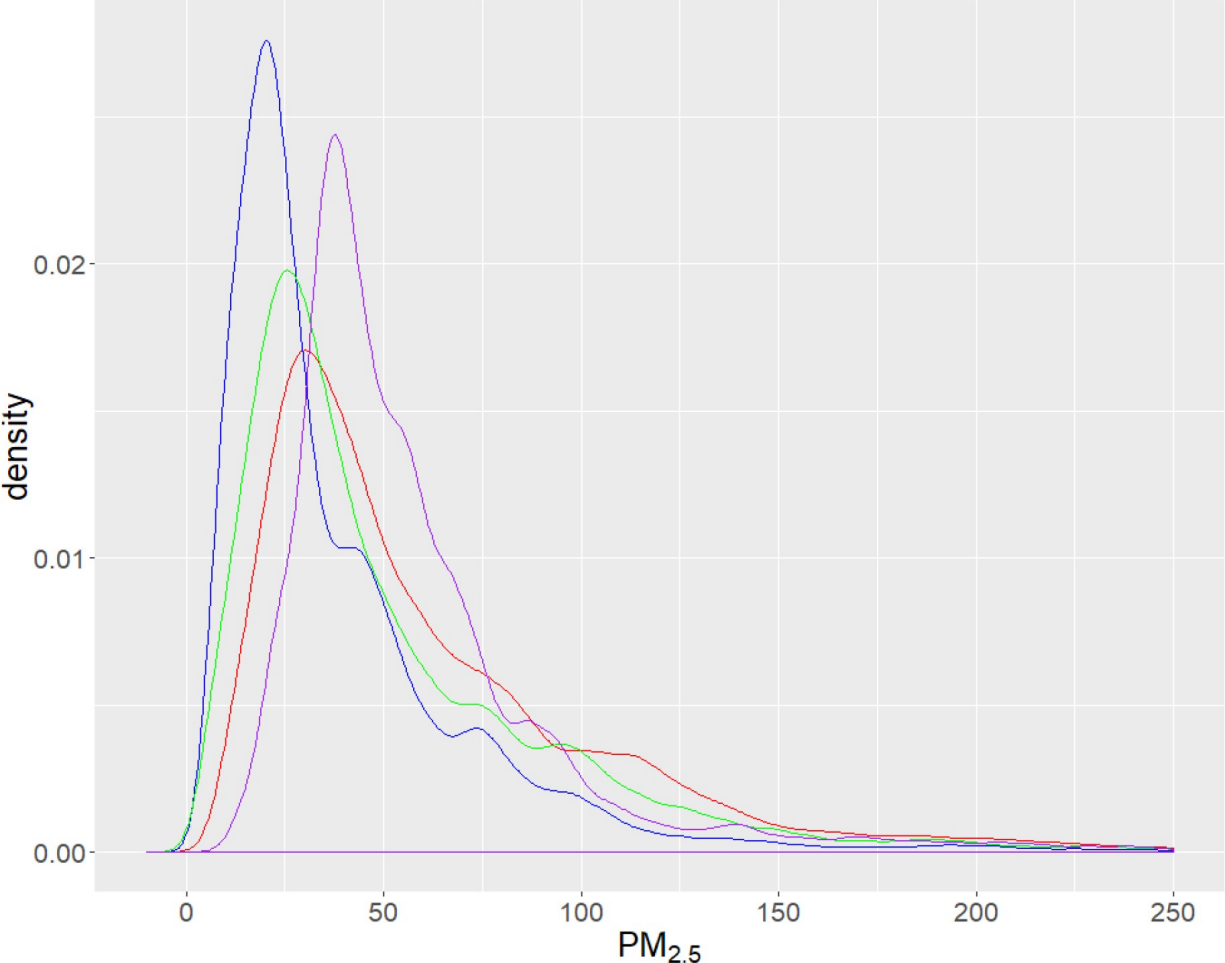
Density plots of hourly PM_2.5_ measurements (μg m^-3^) at street level in Liverpool, western Sydney. Red (sensor AQ2) in pedestrian mall (intersection Macquarie St mall & Elizabeth St), Blue (AQ6) intersection of Moore St & Bigge St, Green (AQ7) Northumberland St, a 2 lane road with a multi-storey car park, between Memorial Ave & Moore St), Purple (AQ9) T–intersection of Bigge St & Scott St, near train station car park.

Readings from other roadside devices (Fig 8) around the city centre showed that elevated readings were common. Even though all devices were located in the city centre, readings differed considerably between locations. This data will allow for relative estimates of exposure at different locations and assist in prioritising where efforts need to be made to reduce exposure to hazardous pollution.

**Fig 9:**
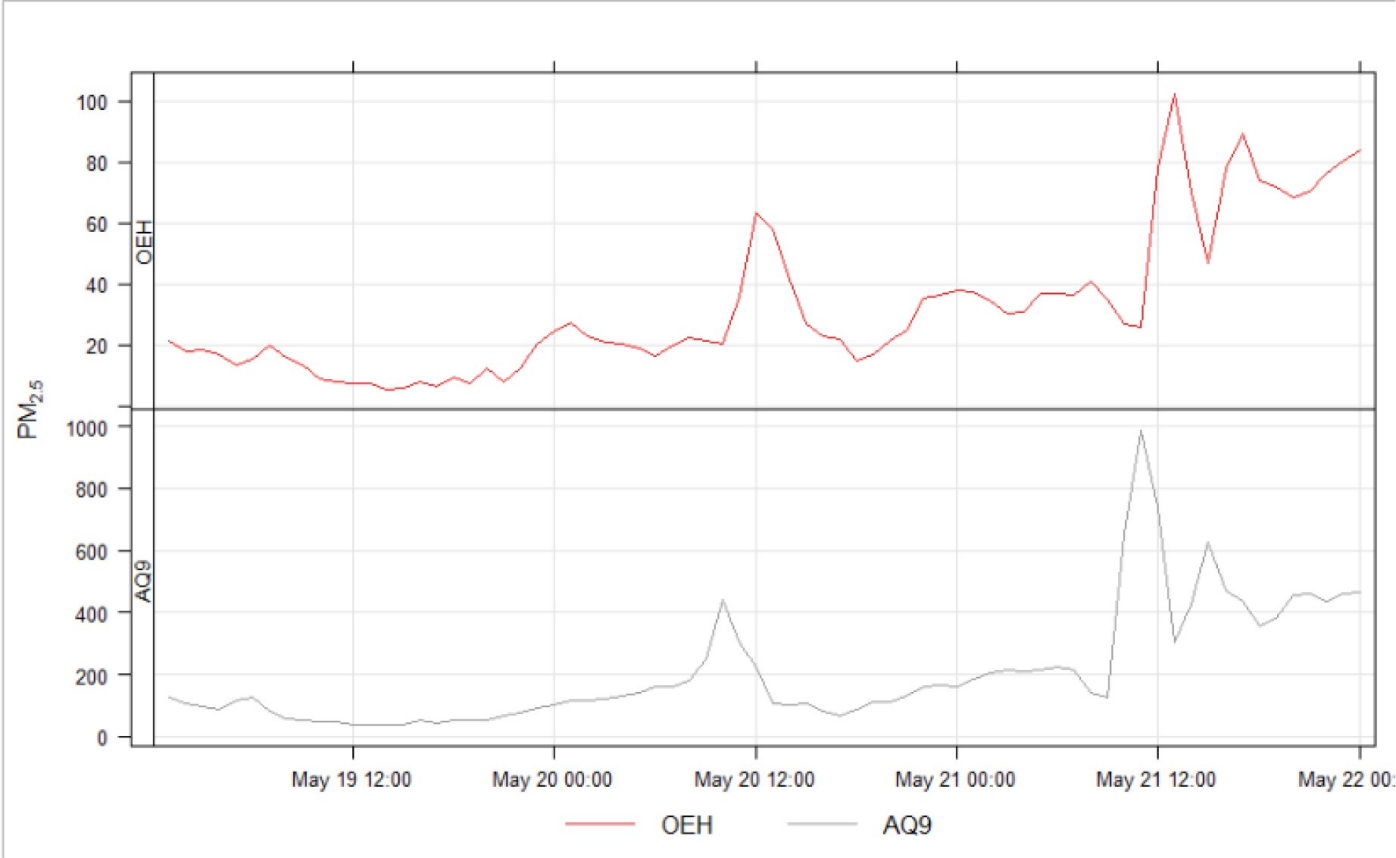
PM_2.5_ data during smoke event due to hazard reduction burn in May 2019, showing elevated concentrations at the roadside. Department of Planning, Industry and Environment (DPIE) Liverpool Air Quality Monitoring Station and roadside sensor AQ9, at the corner of Bigge and Scott Streets (for map, see Fig 1).

The roadside devices detected a strong signal during smoke events from bush fires (Fig 9). The timing was consistent with the DPIE equipment, even though, under ambient conditions (no fires), the daily trace for roadside sensors had shown little relation to the Air Quality Monitoring Station. During these events, measurements of smoke were consistent between the 2 locations, but concentrations were much greater at street level. This pattern was consistent for other smoke events.

## 4. Discussion

PM_2.5_ is an important parameter to measure in air quality monitoring, due to its implications for health in cities and regulatory requirements. There are a number of different technologies used to make the measurement and they may give different readings for the same concentration of different types of PM_2.5_, since particles can exhibit different shapes and composition. The only method currently used in low–cost sensors is optical scattering. This is also found in more expensive equipment and these are used for a wide variety of types of PM_2.5_. It is a relatively stable technology that can be manufactured to reasonable standards of accuracy and consistency in mass production. There are increasing numbers of low-cost optical scattering units on the market, due to the boom in wireless connected sensors (Internet of Things); unfortunately, these are not all of a useful quality.

We tested several different sensors and found problems ranging from loss of sensitivity over time, to a design where the inlet & exhaust were close to each other. The best performing low-cost units at the time of writing were NovaSense SDS011, consistent with the conclusions of other recent studies [46, 47]. These were found to have excellent repeatability, giving consistent readings between different units in the same housing. Tests of the sensors alongside reference quality instruments yielded good results over an extended deployment. There was excellent agreement between the reference equipment and the low-cost devices in Wollongong, during the episode of smoke pollution from bushfires near Avon Dam, NSW.

A network of sensors installed in the streets around Liverpool, western Sydney yielded information about particle pollution in busy pedestrian precincts that was not otherwise available. Concentrations at roadsides were often up to an order of magnitude greater than background values measured at a nearby air quality station. Since the publicly available air quality data is derived from the background readings, roadside data provides valuable additional information for people spending time in city streets. Accuracy of PM_2.5_ measurements may be improved with the addition of sensors to measure temperature and humidity [46].

The devices were reliable, after initial software bugs and hardware problems were eliminated (e.g. communications problems, drifting of the real-time clocks, power management) and the housing optimized for outdoor environments. Those attached to mains power and connected to the LoRaWAN network returned data 95% of the time. The only downtimes were due to an outage of the network infrastructure used by The Things Network. Since the sensors are transmitting a limited amount of information every 40 seconds (the payload of each message is only 12 bytes), the sensors have a very limited footprint on the overall capacity of the network.

The network of sensors has been deployed for over a year; we do not know how well the sensors will perform over extended periods. It is likely that at some stage, continuous exposure to contaminated air will result in a loss of sensitivity. We plan to re-test the performance of the sensors following the recent extended and severe bushfire season. Finally, as part of the Smart Cities, Smart Liverpool, Smart Pedestrians project, air quality sensors are collocated with other visual sensors dedicated to monitor the flow of people and vehicles. The data from the two types of sensors can be combined to compute the PM exposure of pedestrians in the central business district.

## Supporting information

S1 Appendix (PDF)
