# Supplementary material for: Traffic exhaust to wildfires: PM2.5 measurements with fixed and portable, low-cost LoRaWAN-connected sensors
Source: PLoS One. 2020 Apr 24;15(4):e0231778. doi: 10.1371/journal.pone.0231778 (PMC7182254; doi:10.1371/journal.pone.0231778)
Supplement: S1 Appendix — (PDF) [file pone.0231778.s001.pdf]

Assembling a SMART Air Quality Sensor

This guide shows how to assemble an air quality sensor, using the LoPy4 chip and other components readily available from local & on-line stores. All images C. Dunn, except where labelled otherwise.

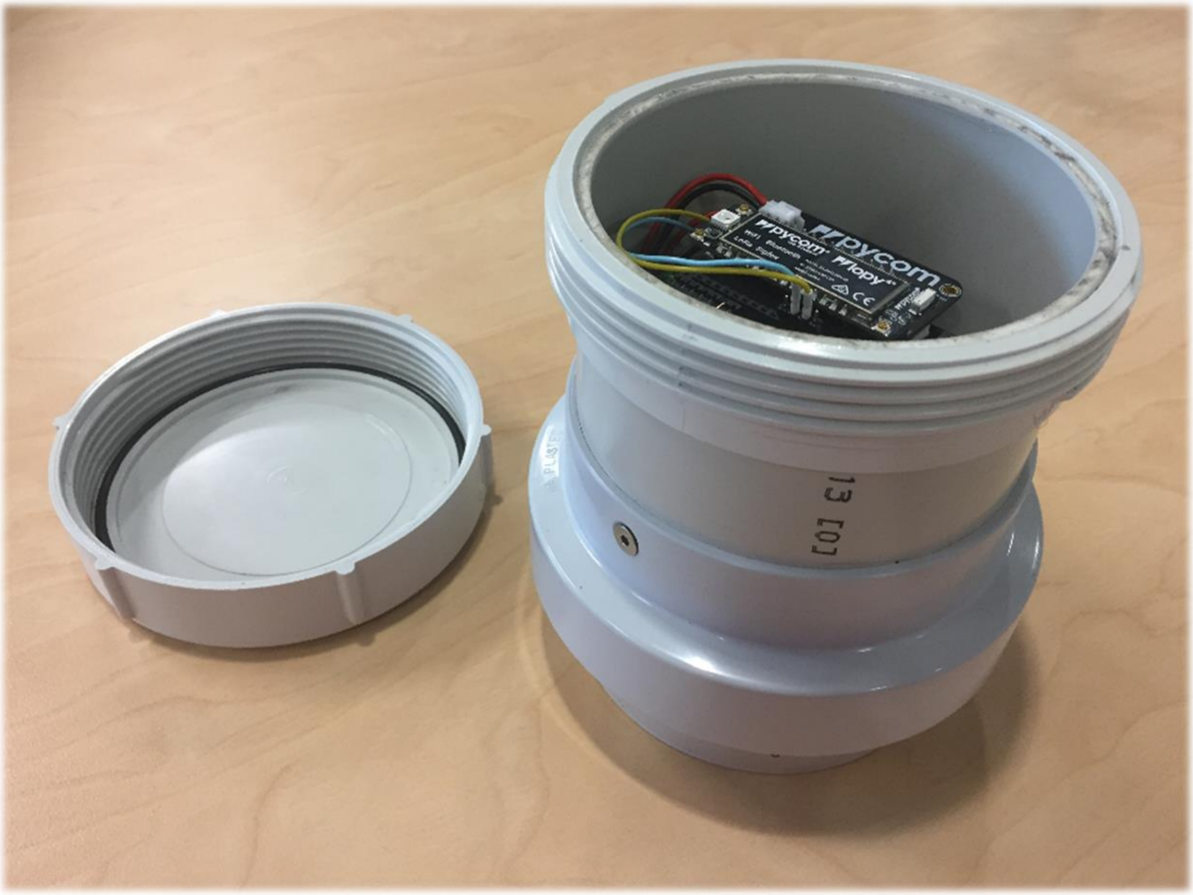

There are three main stages in the assembly.

1 – Assembling the Air Quality Sensor Housing.

2 – Assembling the Air Quality Sensor Internal Components.

3 – Putting it all together.

Each step is explained in detail in the remainder of this guide. Please make yourself familiar with the entirety of each assembly stage before commencing the construction.

At the end of this guide (p21) are Scale drawings, that shows the exact measurements and dimensions of various cuts and holes that will need to be made.

Assembling the Air Quality Sensor Housing

The main components are shown below:

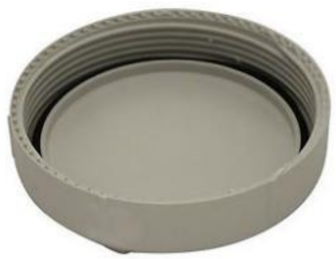

A – 100mm PVC DWV Threaded Access Cap

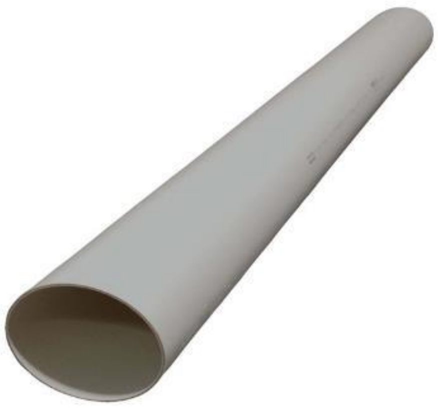

D – 100mm & 40mm PVC DWV Pipe

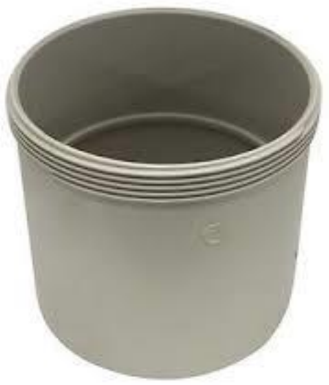

B – 100mm PVC DWV Threaded Access Coupling

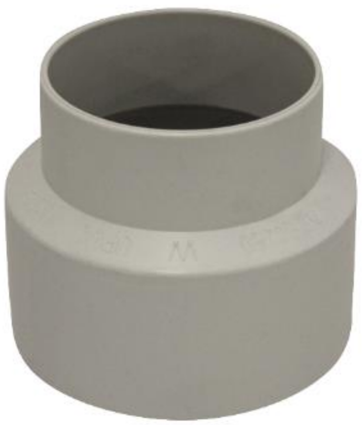

E – 100mm PVC DWV Weathering Apron

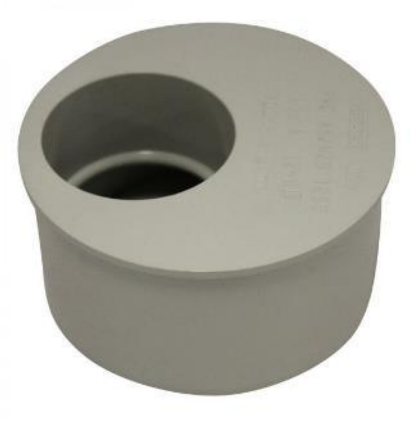

C – 100mm X 40mm PVC DVW Pipe Reducer

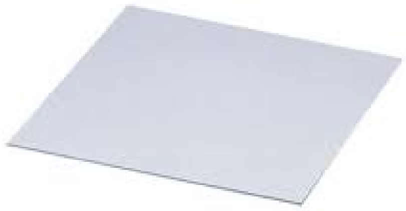

F – 3mm thick PVC sheet

Some of these components need to be modified before construction. See Scale drawings (p21) for a diagram showing exact measurements in orthogonal projection view.

In addition to these components, you will also require 5x flat bottomed, self-tapping screws, PVC plumbing adhesive, and some form of fine mesh netting (fibreglass fly screen is ideal).

Modifying the components

B – Reduced to 30mm in length, preserving the thread.

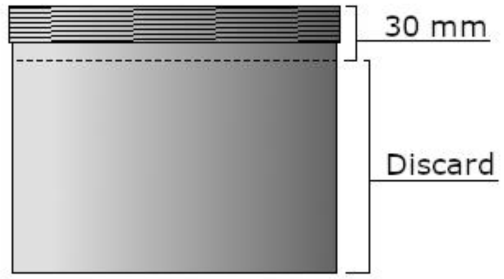

D – Cut three rings of 140mm, 21mm, and 16mm.

Cut the pipe into rings as shown below. The 140mm section **becomes part D** and will be used as the main part of the Sensor housing. The 21mm and 16mm sections are used on the inside of the housing to brace other components. . Left-over parts of pipe can be kept for spare, in case of errors in construction.

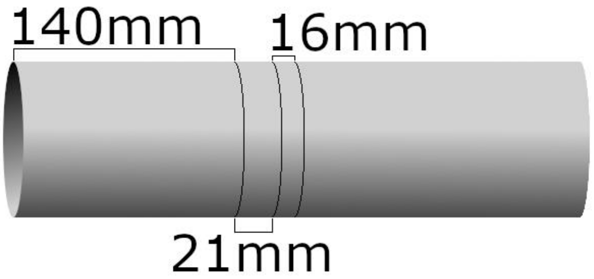

Cut a 10mm wide slice out of each of the two smaller rings. This will allow the rings to compress slightly so that they can be placed inside D. Mark a line around D, 52.5 mm from the bottom. Mark and drill 21 x 12mm holes, equally spaced along the line. These holes will allow air to flow through the housing.

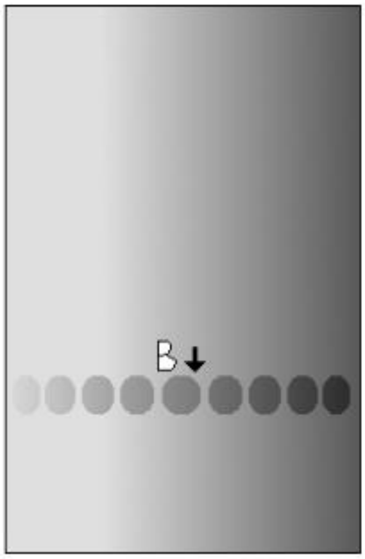

E – Reduce E to 44mm high and pre-drill screw holes

Only the top part of the weathering apron is used. Cut off at 44mm as shown and recycle the lower part. Drill 3 equidistant holes as shown in Scale drawings (p21). The holes are to accommodate screws in a later step. Ensure the holes are suitable for the screws you will use.

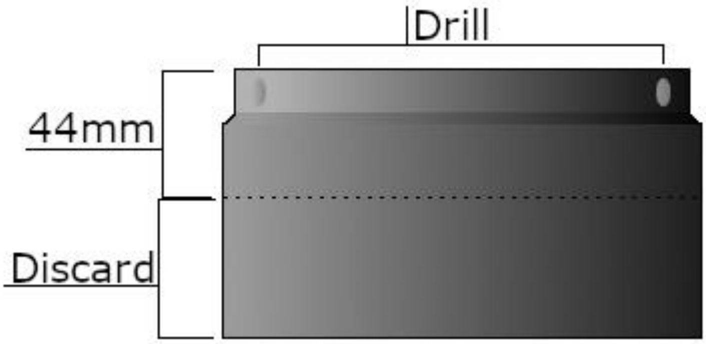

C – Trim to prepare for the fan.

In order to accommodate the fan, parts of C need to be cut away. See the circled sections, below. Test the fit with the fan and adjust before starting the assembly.

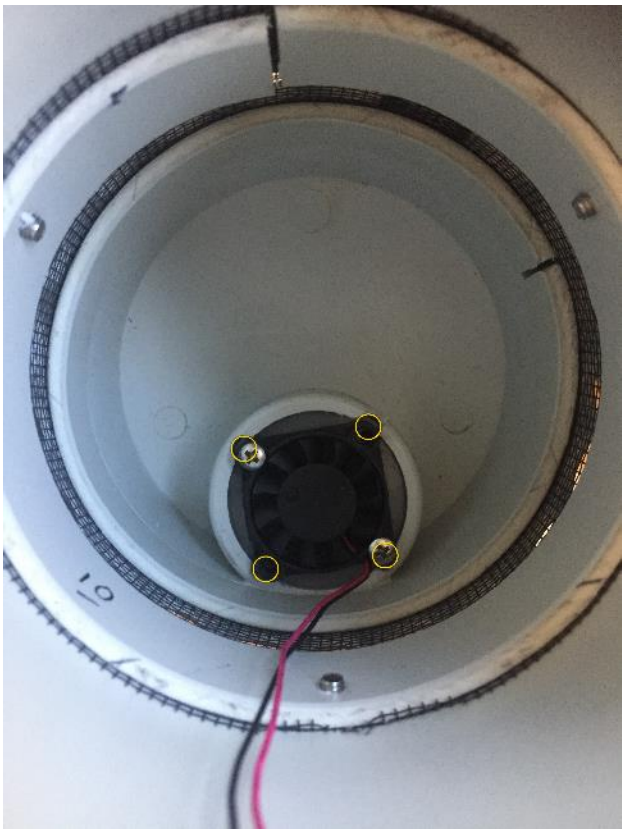

Cut the outside of the reducer (but not the 40 mm inset) at 25 mm from the closed end:

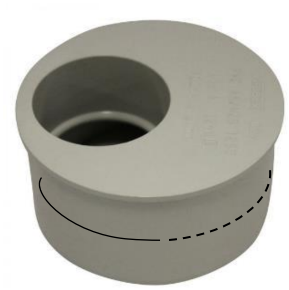

Drill a 1mm hole in the base of C. This is to allow condensation to drain from the device. Drill another hole near the 40m inlet for the power cable.

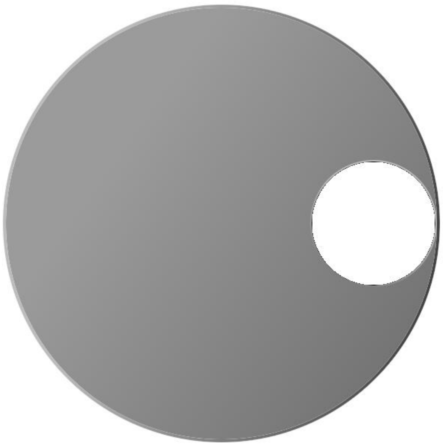

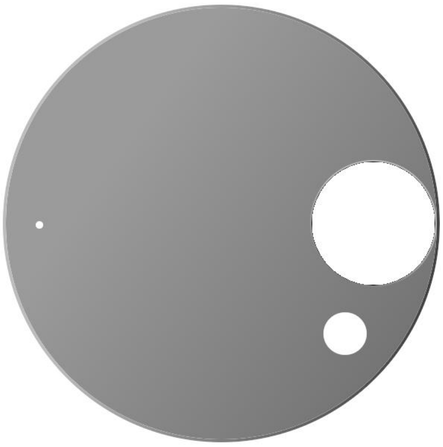

Assembling the components

Use adhesive to attach C to D, as shown.

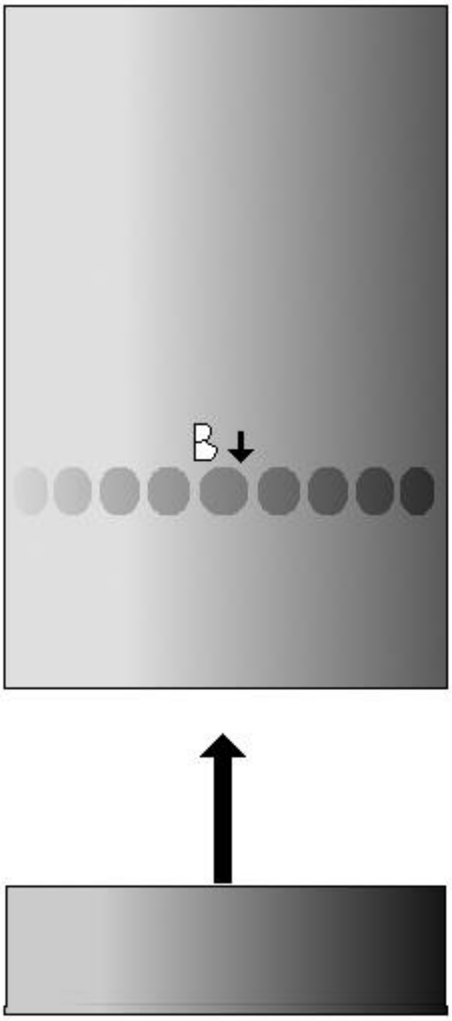

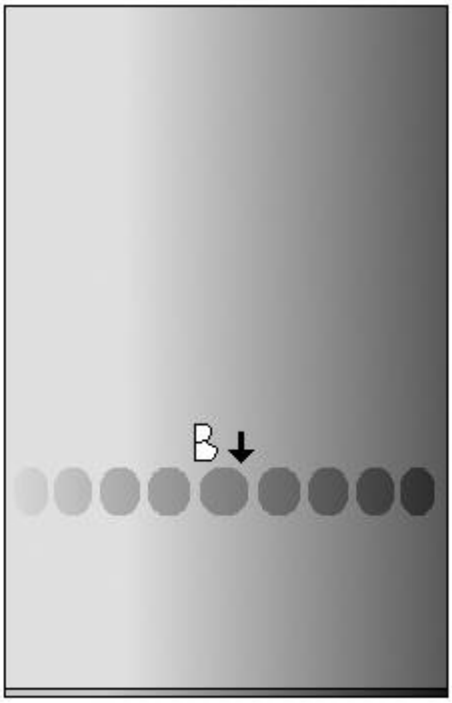

C will fit snugly inside D.

Take the mesh and prepare a 312mm X 45mm section. Place it inside D and resting against the top of C, as shown below. Use the 16mm ring section from D to hold the mesh in place.

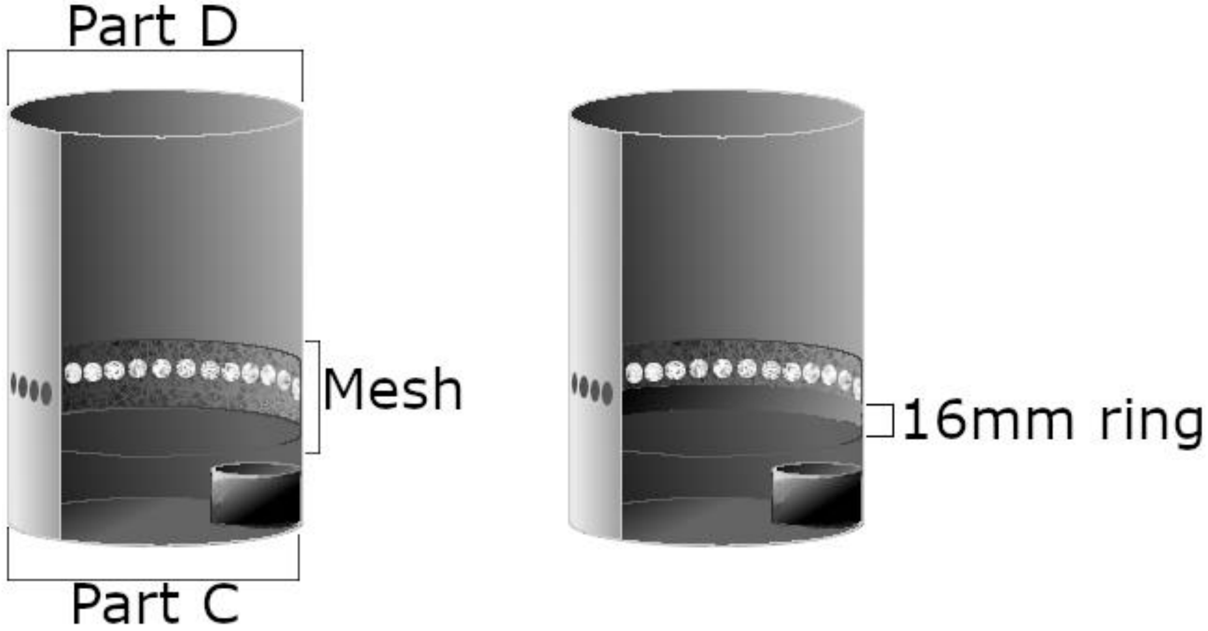

Place E over D, aligning E so that it is 70mm from the bottom of D, as shown. E protects the holes in D from rain, while allowing air flow. Mark through the holes you pre-drilled through E on to D, then remove E. Then drill (on D) the marks you just drew.

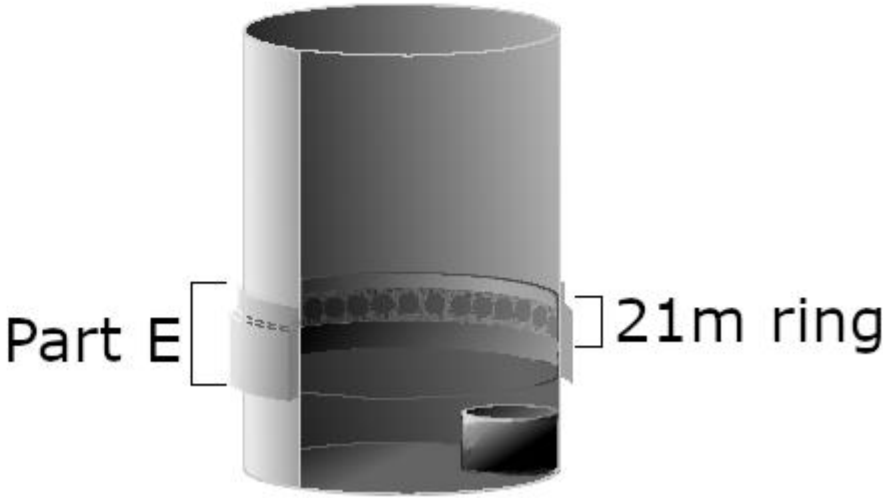

Temporarily affix the 21mm ring into position with a clamp or brace. Using the holes drilled into D, mark up the 21mm ring, and then remove the ring. Drill through these marked areas.

Replace the 21mm ring on the interior of the housing, drill & set screws on opposite sides of D, through E, D and the 21mm ring. Screws should be self-tapping, with a flat head.

Drill & set another screw through D and C, 10 mm above the base, lining up the screw to penetrate the inner 40mm tube. See Scale drawings (p21) for the exact placement of these screws.

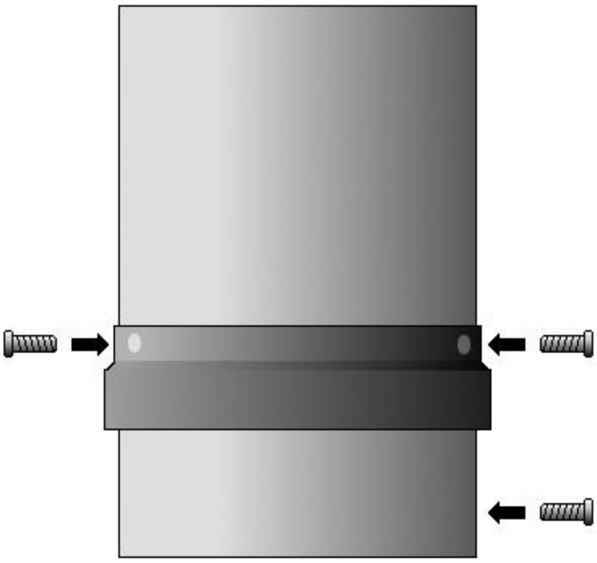

Apply a contact adhesive to the interior of B, and attach it to the top side of D, so that the thread extends just above D, so the lid will seal against B.

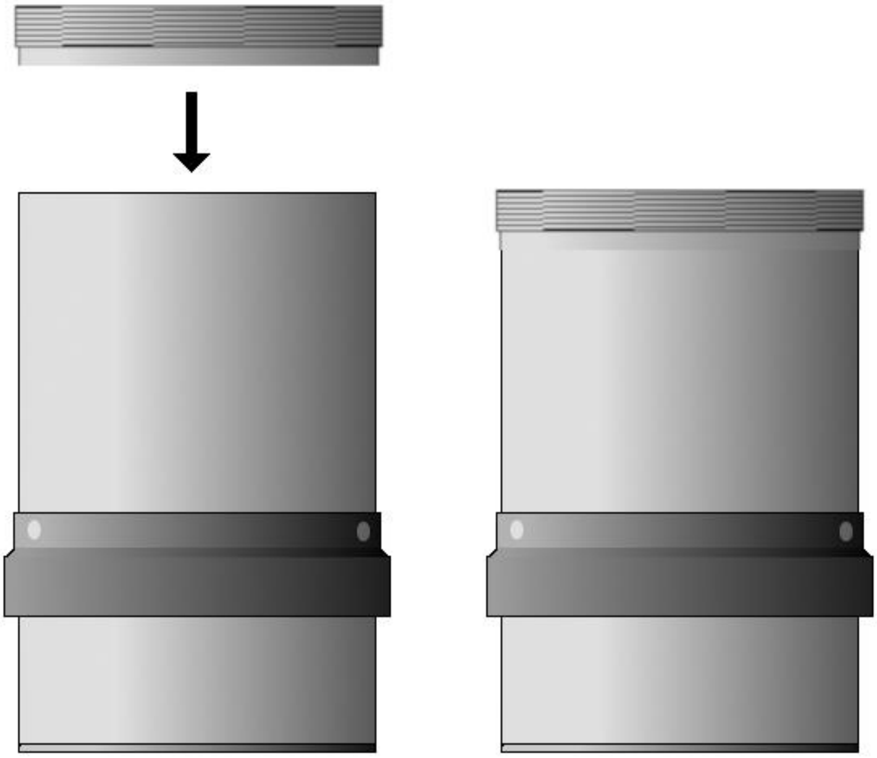

9 – The Sensor Housing is complete. Set aside and allow to dry.

Assembling the Air Quality Sensor Internal Components

You will need the following components:

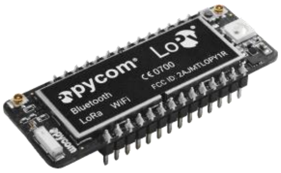

A – Pycom LoPy4 chip

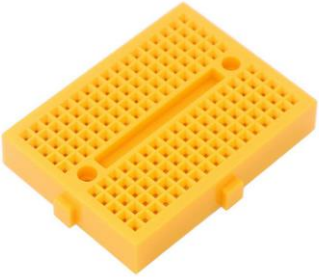

B – A mini breadboard (35 x 45 mm)

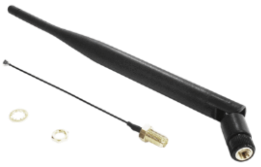

C – Pycom LoPy Aerial

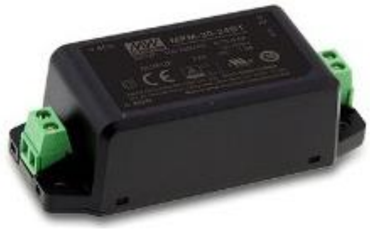

D – MPM-30-5ST Transformer

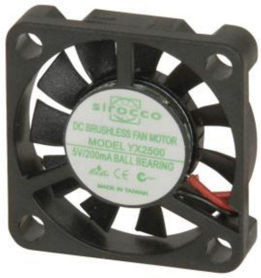

E – Sirocco YX2500 5V DC 30mm brushless rotor fan

F – JST connector and cable

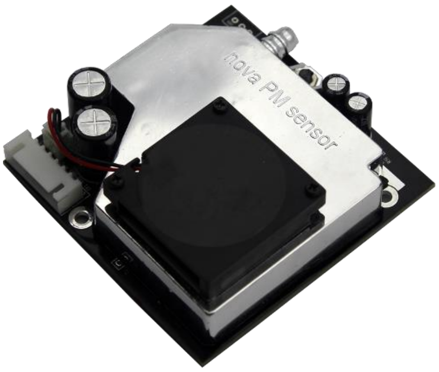

G – Nova PM Sensor

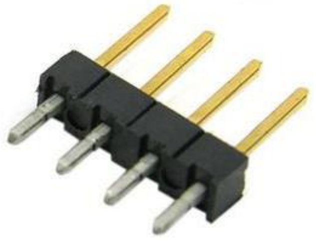

H – 2x Male 4-pin Single Row Breakaway Header

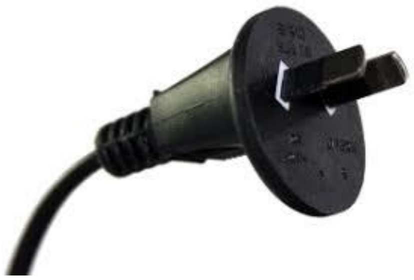

I – Standard AC power cable

Other miscellaneous components:

A waterproof cable gland to affix through the base of the device to accommodate the power cable.

Four lengths of wire (about 150 mm) a small amount of solder.

Double sided foam tape (at least 25mm x 45mm), to attach the electronics to the transformer

3 pieces of 3 mm PVC sheet:

- 90mm diameter disc,
- 40 mm disc
- 70 mm square

Assembling the components

Mark a chord 70mm in length across one edge of the 90 mm disc cut along the line (1 below). Drill a hole through a corner of the disk as shown in 3, below, this is to accommodate cables. Use plastic welding to attach the 70mm square at a right angle, on the inside of the straight edge (otherwise the platform will not fit in the housing).

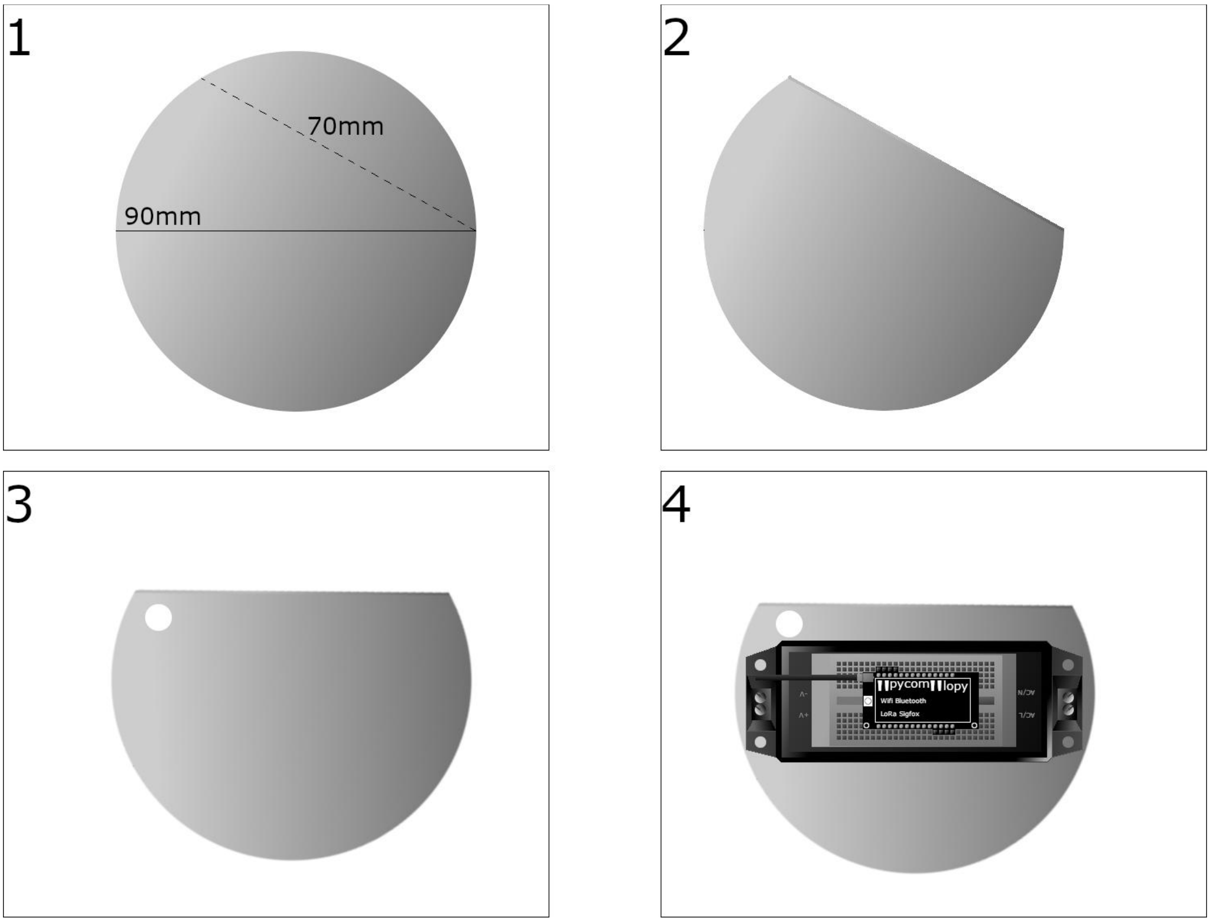

Set up the LoPy first- an expansion board is needed initially to provide a USB connection to a computer. Update firmware, then add code from GitHub.

Flash the software to the LoPy4 using an expansion board and a program such as Atom with the Pymakr package (freely available). The code is available at: https://github.com/jojo-/air-quality-lopy

If you cannot connect to your device, it may need the firmware to be updated. Instructions to update the firmware can be found at:

https://docs.pycom.io/gettingstarted/installation/firmwaretool

Starting with the mini-breadboard (1), attach the LoPy4 (2). Add one breakaway header (part H) on each side of the LoPy4 as shown in Scale drawings (p21) (3). Attach the Aerial (Part C) to the LoPy4, taking care to attach the aerial to the correct port as shown in (4). This is important as you may permanently damage the LoPy4 if you attempt to use it without an aerial attached.

1

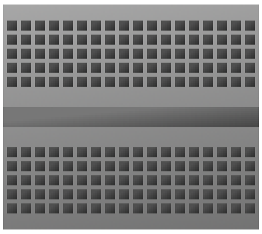

2

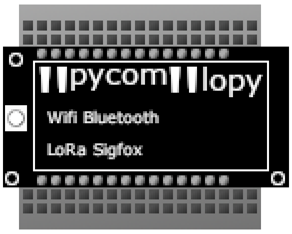

3

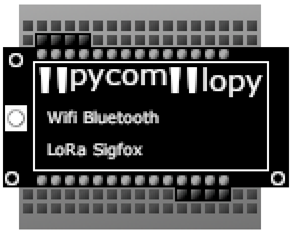

4

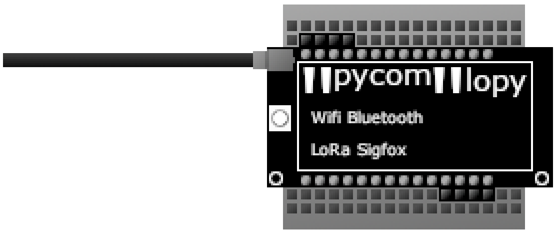

Take the transformer (part D) and check the writing on the top. The terminals on one end is the mains supply input, AC/L (240 v alternating current/live) and AC/N (240 v alternating current/neutral) terminals, and the other has the output 5 v DC positive and negative terminals. The top of part D indicates which terminal is which, take care not to obscure the text for the output terminals in the next step. Also take a marker, and indicate on the terminal blocks which terminal is which before proceeding. The positive/negative terminals should be on your left when the LoPy is added on top. See below for the correct orientation.

The correct orientation is critical.

One end will be receiving a 240v mains current, the other will be giving out a 5v current.

The transformer may be destroyed if connected in the wrong orientation, the LoPy may be destroyed if the polarity of the output voltage is reversed.

Either use the double-sided tape included with the breadboard, or cut a strip of 3M double sided foam tape and affix it to the top of part D, and then place the breadboard on top of this tape. Make sure the A/C terminal is on the opposite side to the aerial.

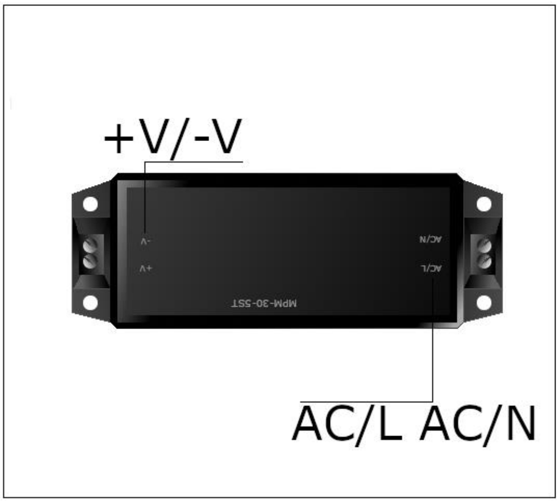
+V/-V
AC/L AC/N

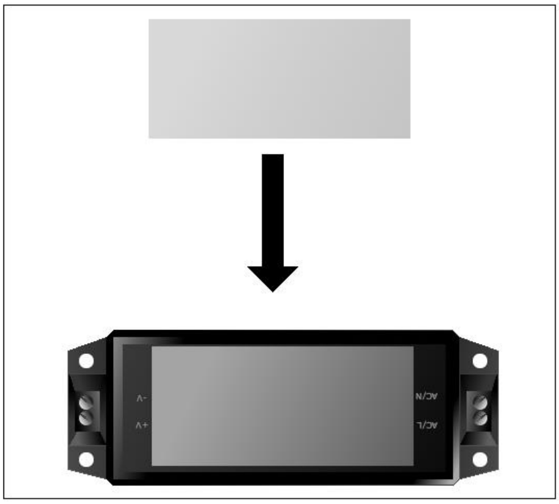

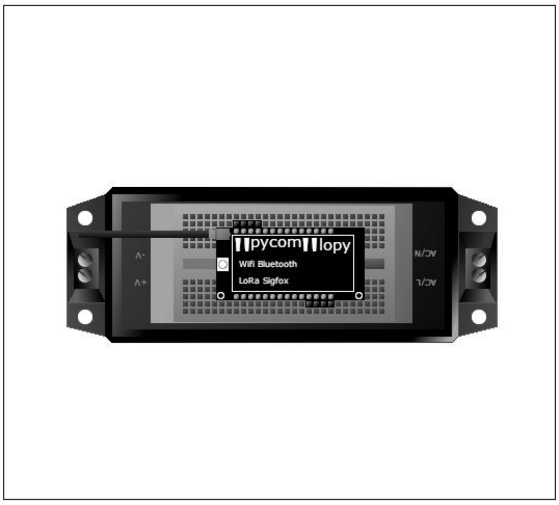
pycom
lopy
Wifi Bluetooth
LoRa Sigfox

Affix the transformer and sensor bundle to the sheet using either another 3M double sided foam, or by using small bolts through the transformer's feet.

Place the transformer as far as you can away from the edge that was cut so that you have room to place the sensor (part G) in a later step. Take care to note the orientation of the text on the LoPy against the section of the disc that was removed. Make sure no part of the transformer is extending outside of the disc, otherwise it won't fit in the sensor housing.

Take the 70mm square of plastic and attach it at a ninety degree angle to the disc of plastic (image 3 below) using a plastic welder.

Run the AC cable through the hole drilled in the bottom of the housing, and affix the waterproof gland to secure the cable. Connect the cable to the AC/L and AC/N side of the transformer. You will need to strip the cable to access the inner cords.

DANGER. Do not connect the cable while you are working.

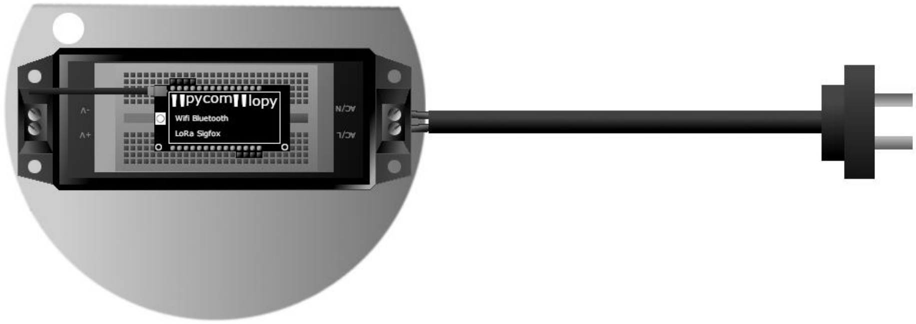

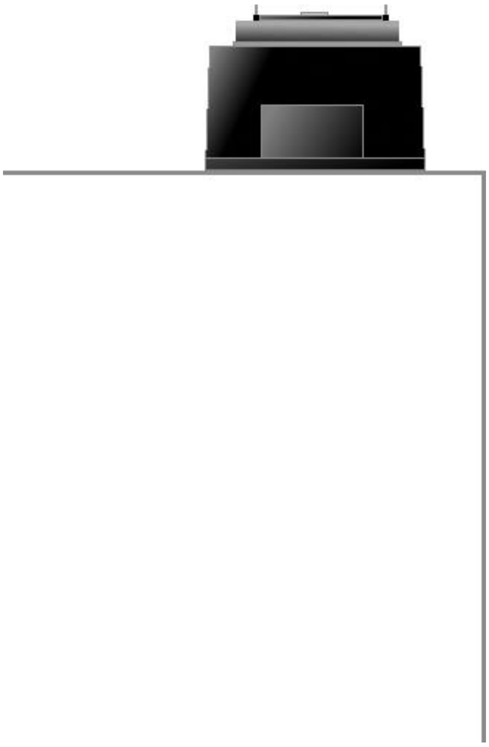

Take the Nova sensor and look underneath. You will need to solder four cables to the TXT, RXD, GND and 5V points. Perform the soldering now, but don't connect the 39/20/GND/5V connections just yet.

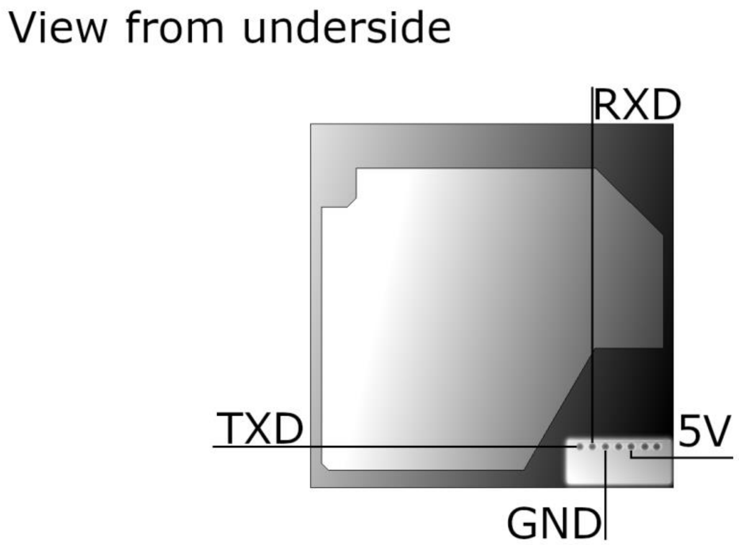

Solder breadboard cables to RXD, TXD, GND and 5V.

Affix the sensor to the 70mm square of plastic sheet, with the underside facing the plastic. You will need to use tubular spacers for the screws in order to leave room under the sensor for the soldered nodes to fit (see picture below).

You can use the hole pre-drilled in the 90mm disk to pass the cables through.

The TXT and RXT points will connect to the 39 and 20 points on the LoPy, while the GND and 5V will be connected to the – V and + V terminals of the transformerGo ahead and join all the cables up, using the following images for reference:

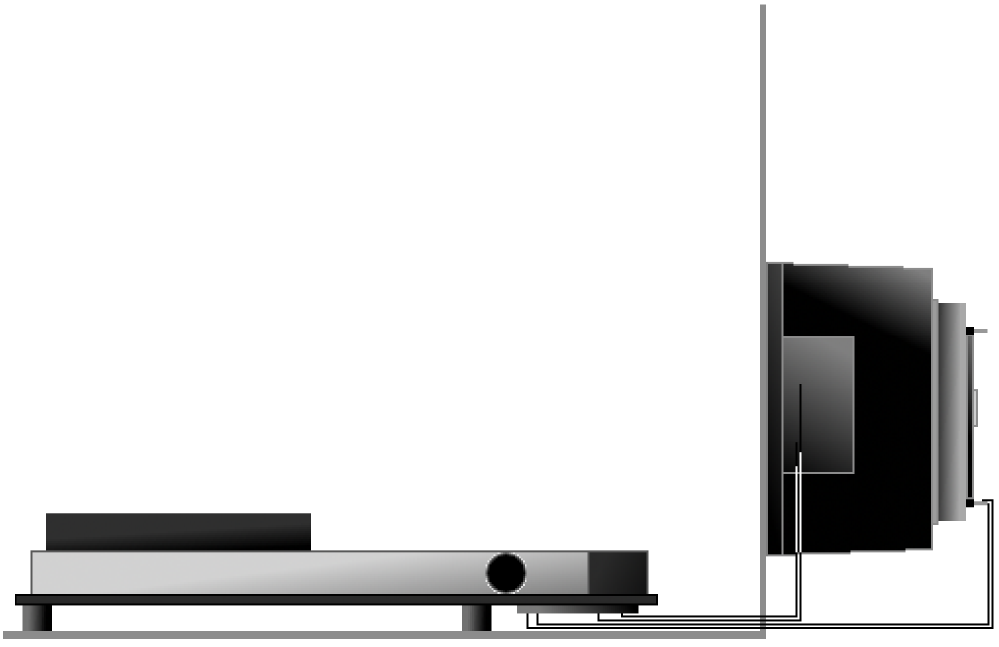

39 to RXD, 20 to TXD. For details and pinout diagram, see the PyCom website:
https://docs.pycom.io/datasheets/development/lopy4
https://docs.pycom.io/gettingstarted/connection/lopy4/

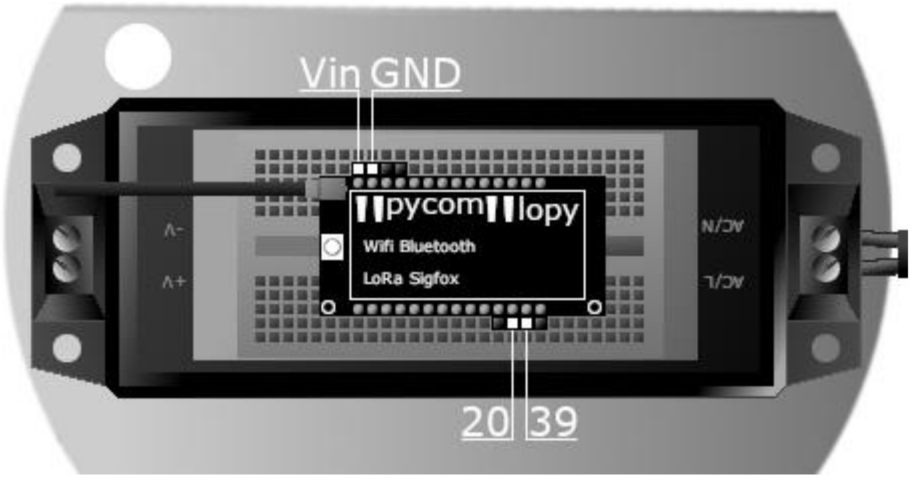

Vin joins to the transformer +V, and GND (from the LoPy) connects to the transformer –V.

The fan is assembled over a few steps.

Start by taking a square of plastic, anchoring it well, and drilling a 35mm hole through the centre. Make this into a ring by cutting around the hole with a 40mm hole saw.

Affix the fan over the disc you have made. Ensure the label is facing outwards.

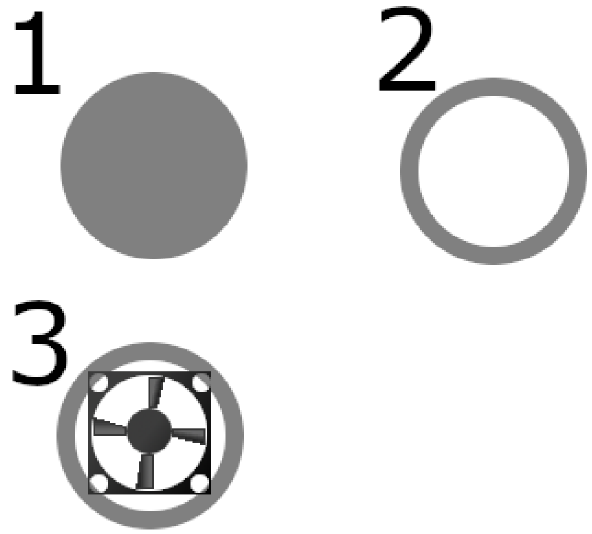

Cut a 50mm diameter circle section of mesh.

From the 40mm pipe, cut a 23mm section. Place the mesh over the top of the 23mm pipe section, and then slide this up the 40mm inlet at the bottom of the sensor housing. This will trap the mesh tightly across the underside of the fan.

Drill another screw through the outside of the sensor housing and into the 40mm section of pipe that was just inserted.

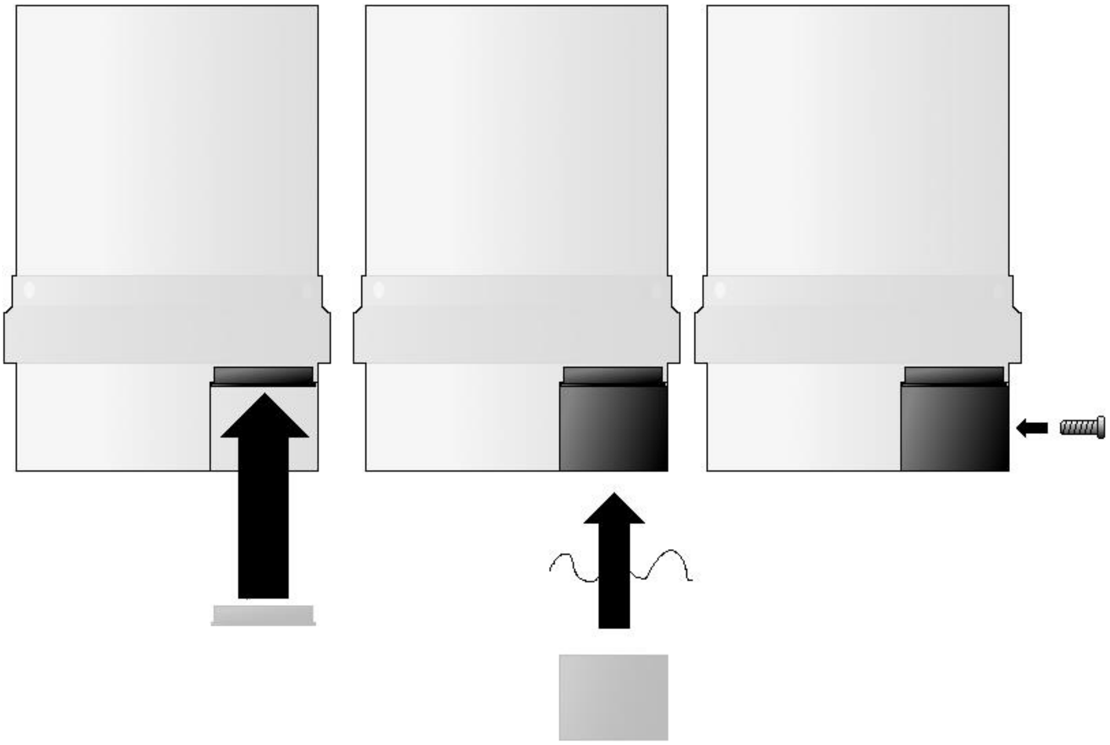

Installing the sensor within the sensor housing

Congratulations, you’re done.

Gently place the sensor within its housing. Make sure the square section of plastic the Nova sensor is affixed to reaches all the way to the bottom and is not resting on the fan.

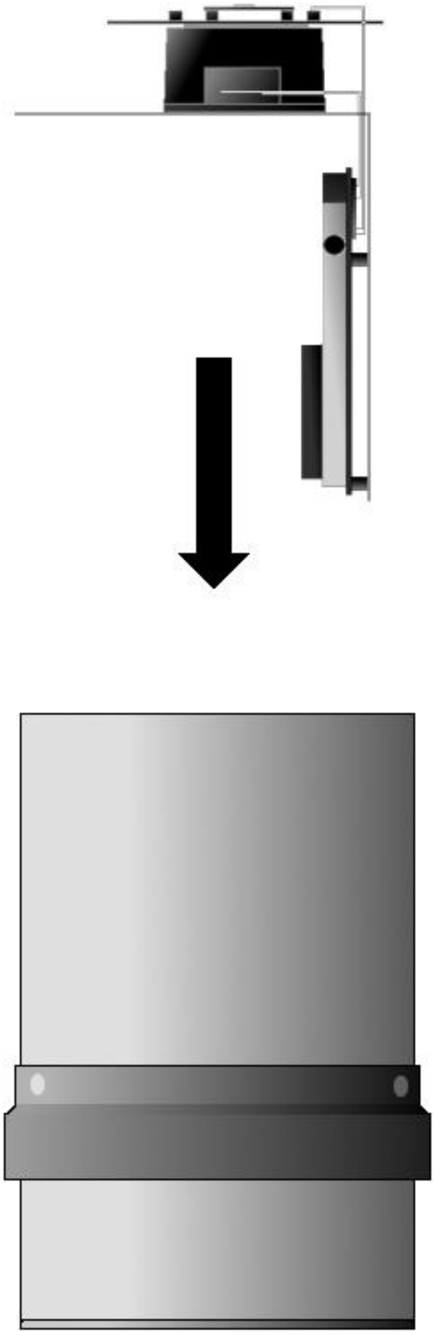

Screw the cap on the housing, and find somewhere to install your sensor!

Scale drawings

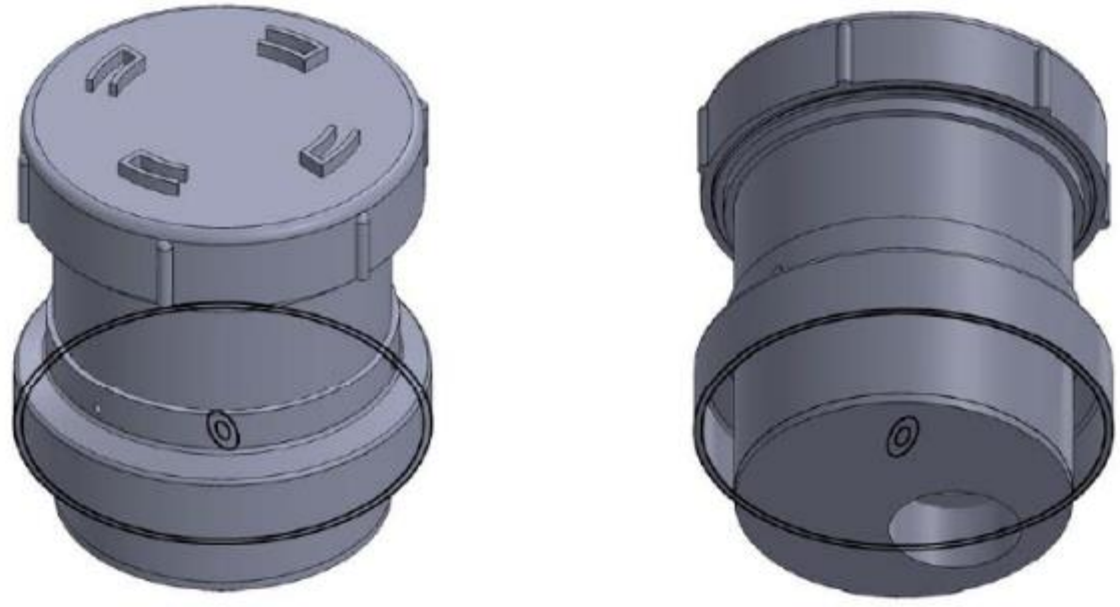

Completed sensor (external view with partial transparency). Image: S. Selby

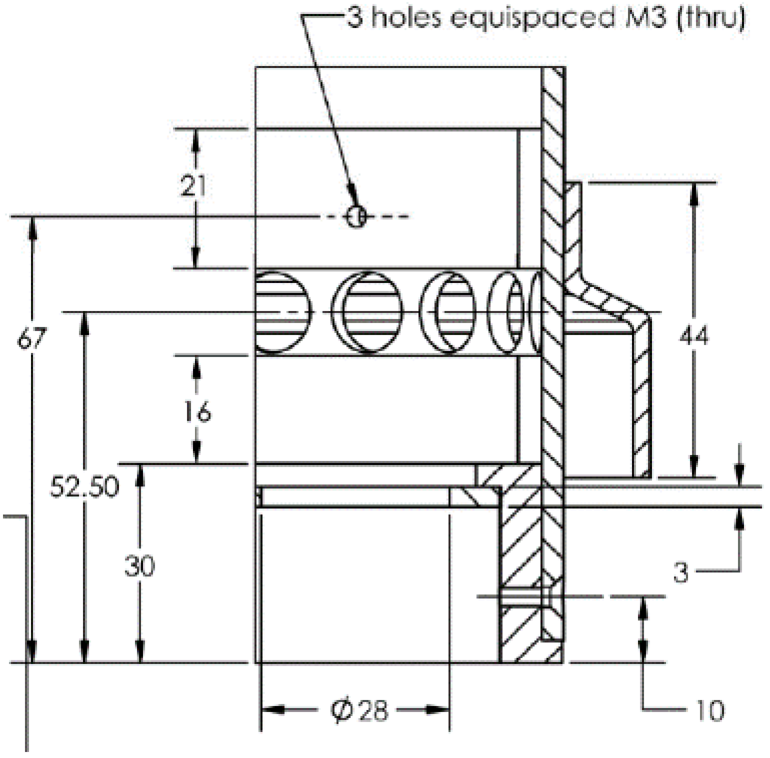

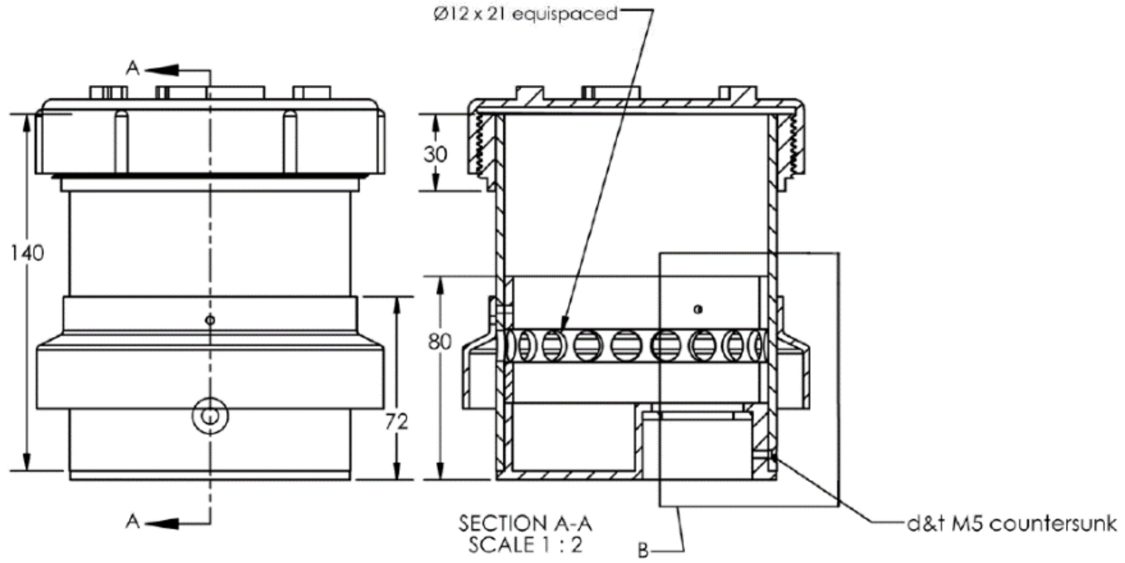

Orthagonal Projection and cross section. Image: S. Selby
